# Efficacy of traditional Chinese medicine *versus* angiotensin-converting enzyme inhibitors, angiotensin receptor blockers, and their combinations in the treatment of IgA nephropathy: a systematic review and network meta-analysis

**DOI:** 10.3389/fphar.2024.1374377

**Published:** 2024-03-21

**Authors:** Sijia Ma, Yuhua Jiang, Linlin Qian, Meng Wang, Shijie Xu, Guowei Wang

**Affiliations:** Institute of Basic Theory of Traditional Chinese Medicine, China Academy of Chinese Medical Sciences, Beijing, China

**Keywords:** IgA nephropathy, traditional Chinese medicine, angiotensin receptor blockers, angiotensin-converting enzyme inhibitors, network meta-analysis

## Abstract

**Background:**

IgA nephropathy (IgAN), a condition posing a significant threat to public health, currently lacks a specific treatment protocol. Research has underscored the potential benefits of traditional Chinese medicine (TCM) for treating IgAN. Nevertheless, the effectiveness of various intervention strategies, such as combining TCM with angiotensin-converting enzyme inhibitors (ACEIs) or angiotensin II receptor blockers (ARBs), lacks a comprehensive systematic comparison. Therefore, this study aimed to conduct a network meta-analysis to assess the clinical efficacy of ACEIs, ARBs, TCM, and their combinations in treating IgAN to offer novel insights and approaches for the clinical management of IgAN.

**Methods:**

A systematic review conducted until November 2023 included relevant literature from databases such as PubMed, Embase, Cochrane, Web of Science, Scopus, CNKI, and Wanfang. Two independent researchers screened and assessed the data for quality. Network and traditional meta-analyses were performed using Stata 18.0 and RevMan 5.3 software, respectively. Outcome measures included 24-h urinary protein quantification (24 hpro), estimated glomerular filtration rate (eGFR), serum creatinine (Scr), blood urea nitrogen (BUN), and adverse event incidence rates (ADRs). Forest plots, cumulative ranking probability curves (SUCRA), and funnel plots generated using Stata 18.0 facilitated a comprehensive analysis of intervention strategies’ efficacy and safety.

**Results:**

This study included 72 randomized controlled trials, seven interventions, and 7,030 patients. Comparative analysis revealed that ACEI + TCM, ARB + TCM combination therapy, and TCM monotherapy significantly reduced the levels of 24 hpro, eGFR, Scr, and BUN compared to other treatment modalities (*p* < 0.05). TCM monotherapy demonstrated the most favorable efficacy in reducing eGFR levels (SUCRAs: 78%), whereas the combination of ARB + TCM reduced Scr, 24 hpro, and BUN levels (SUCRAs: 85.7%, 95.2%, and 87.6%, respectively), suggesting that ARB + TCM may represent the optimal intervention strategy. No statistically significant differences were observed among the various treatment strategies in terms of ADR (*p* > 0.05).

**Conclusion:**

The combination of ACEI or ARB with TCM demonstrated superior efficacy compared to ACEI/ARB monotherapy in the treatment of IgAN without any significant ADRs. Therefore, combination therapies can be used to enhance therapeutic outcomes based on individual patient circumstances, highlighting the use of TCM as a widely applicable approach in clinical practice.

**Systematic Review Registration:**

https://www.crd.york.ac.uk/PROSPERO/, identifier CRD42023476674.

## 1 Introduction

Immunoglobulin A nephropathy (IgAN) involves the deposition of IgA- or IgA-dominant immunoglobulins in the glomerular mesangial area, leading to a range of clinical manifestations such as hematuria, proteinuria, edema, and hypertension (Matsumoto et al., 2022). The prevalence of IgAN is high in Asia, with most patients experiencing an insidious onset (Wyatt and Julian, 2013). Proteinuria is a common clinical manifestation of IgAN (Xia et al., 2022). Notably, proteinuria is the primary risk factor for renal function deterioration in individuals diagnosed with IgAN (Gadola et al., 2023). Approximately 30%–40% of IgAN cases progress to end-stage renal disease within 10–20 years after diagnosis, necessitating dialysis or kidney transplantation for life-sustaining treatment. This imposes a significant burden on patients, their families, and society (Jarrick et al., 2019; Floege et al., 2022).

The detailed pathogenesis of IgAN remains poorly understood, posing challenges to effective treatment. Some scholars have posited that dysregulation of the mucosal immune system contributes to a deficiency in the galactosylation of IgA, subsequently leading to the development of autoantibodies (IgG) targeting non-galactosylated IgA. Ultimately, the deposition of these IgG–IgA immune complexes within the mesangium culminates in glomerular inflammation, which is the primary underlying mechanism driving the disease (Rajasekaran et al., 2021; Schimpf et al., 2023).

Despite advancements in our understanding of the IgAN pathophysiology, no specific treatment is currently available. Currently, the management of patients with IgAN relies primarily on a uniform therapeutic strategy for all chronic glomerular diseases. The 2012 Kidney Disease: Improving Global Outcomes (KDIGO) guidelines for glomerular disease management emphasize that reducing proteinuria, employing renin-angiotensin receptor blockers, and controlling hypertension are the preferred treatment modalities for IgAN (Inker et al., 2014). This entails utilizing angiotensin-converting enzyme inhibitors (ACEIs) or angiotensin II receptor blockers (ARBs) to suppress the renin-angiotensin system (RAS) for blood pressure regulation and minimize proteinuria to decelerate albuminuric IgAN progression.

After receiving supportive treatment, hormonal and immunosuppressive therapies are commonly administered to patients with IgAN if proteinuria above 1 g/day persists. However, it is important to note that these treatments carry potential risks of adverse reactions, which may impact the overall therapeutic efficacy (Lv et al., 2017). In addition to contemporary drugs, traditional Chinese medicine (TCM) has a long history. Several studies have confirmed the efficacy of Chinese herbal medicines for reducing liver and kidney injury (Samarghandian et al., 2017; Eftekhari et al., 2020). Numerous Chinese studies have confirmed the safety and efficacy of TCM in the treatment of IgAN (Li et al., 2020; Wang et al., 2021b). Several meta-analyses have validated the effects of each treatment modality on IgAN (Zhao et al., 2021b; Huo et al., 2021; Wang et al., 2022). It is imperative to investigate an optimal treatment protocol and leverage the complementary and alternative attributes of TCM to ameliorate symptoms, mitigate toxicity and adverse effects associated with contemporary medical interventions, enhance therapeutic efficacy, and diminish the likelihood of recurrence. However, the effectiveness of different intervention strategies such as combining TCM with ACEI or ARB remains unclear. Therefore, we conducted a network meta-analysis of randomized controlled trials (RCTs) to systematically evaluate the effects of various intervention strategies on key renal outcome indicators in patients with IgAN and provide insights and support for future clinical treatments.

## 2 Materials and methods

### 2.1 Study design

This study was conducted in accordance with the Preferred Reporting Items of Systematic Reviews and Meta-Analyses (PRISMA) guidelines while referencing relevant sources. The study protocol was registered in the International Registry of Prospective Systematic Reviews (PROSPERO) under registration number CRD42023476674.

### 2.2 Search strategy

**FIGURE 1:**
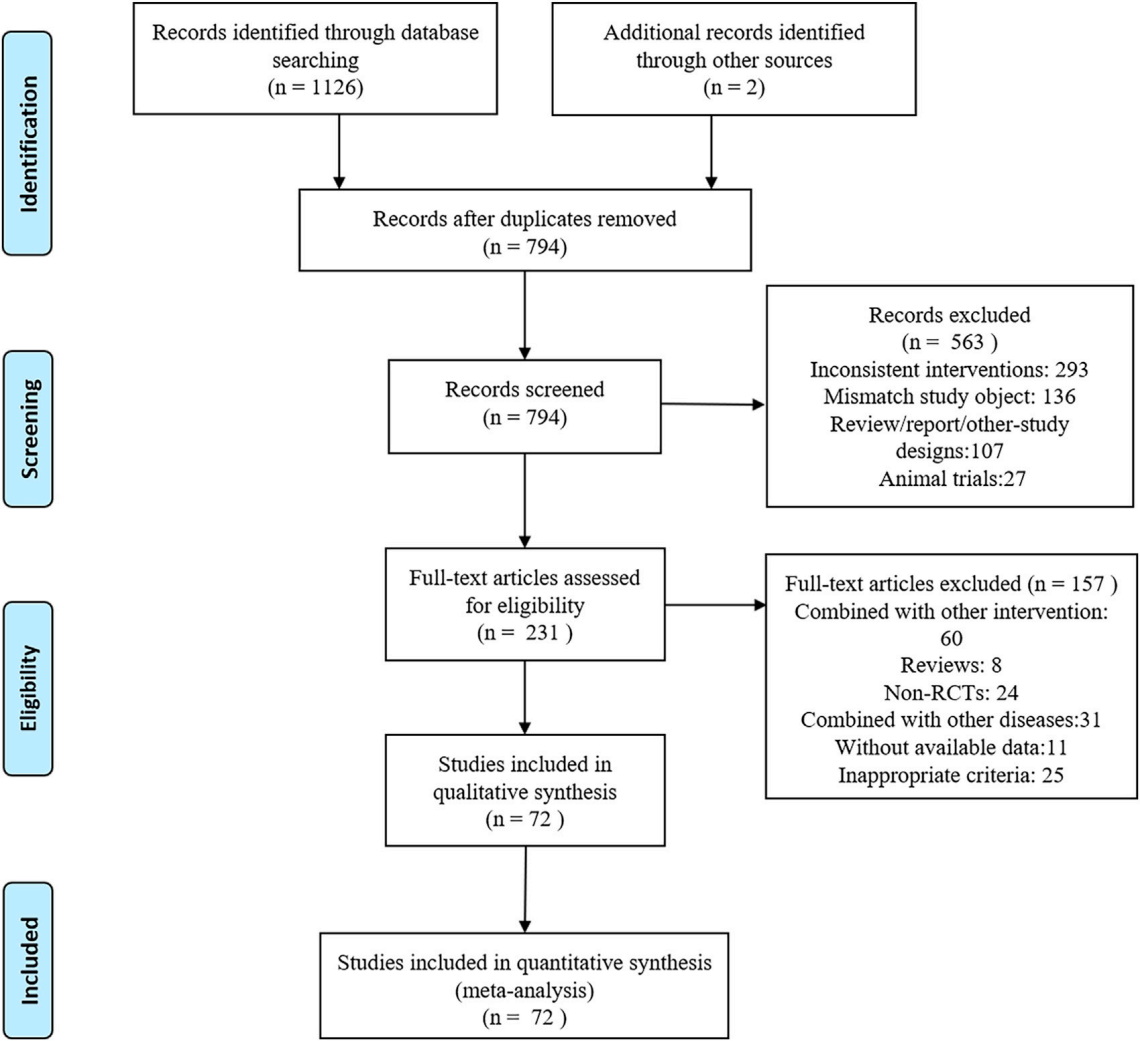
PRISMA flowchart of the screening process for study inclusion. RCT, randomized controlled trial.

Electronic databases including PubMed, Cochrane Library, Scopus, Embase, Web of Science, China National Knowledge Infrastructure, and Wan Fang were searched. The deadline for the search was November 2023 with no language restrictions. Supplementary Table S1 provides further information on the search strategy. The retrieved articles were imported into the Endnote X9 software to eliminate duplicate entries. Two reviewers (SJ Ma and YH Jiang) independently reviewed the titles, keywords, and abstracts of the literature selected in these formats; when they had different opinions, they were discussed with a third reviewer (Linlin Qian) and then an optimal solution was found. Data extracted included the following: title, authorship, diagnostic criteria for the disease, disease stage classification, patient count, average age of the participants, sex distribution, study design type, intervention details, treatment duration, outcome measures employed, and adverse events recorded. The study methodology is illustrated in Figure 1.

### 2.3 Inclusion criteria

Studies were selected for inclusion if they met the following criteria: (1) study design: RCT methodology; (2) participants: adult patients with primary IgAN; (3) intervention: the treatment group received ACEIs, ARBs, TCM, or any appropriate combination thereof, in addition to conventional treatment if necessary, whereas the control group received a single class of the above-mentioned drugs or a placebo along with usual care; (4) outcome measures: estimated glomerular filtration rate (eGFR), 24-h urinary protein (24 hpro), serum creatinine (Scr) levels, blood urea nitrogen (BUN) levels, and adverse drug reactions (ADRs). Only articles published in peer-reviewed journals were included in this review, and both English and Chinese language publications were eligible for inclusion. The studies selected for inclusion had at least one of the aforementioned outcome measures evaluated and a minimum treatment duration of 3 months.

### 2.4 Exclusion criteria

We excluded duplicate publications, studies lacking sufficient data, studies without full texts, studies with unclear observational processes, studies with inconsistent baseline characteristics between the experimental and control groups, and trials involving patients under 18 years of age, secondary IgAN, pregnancy, and treatment duration of less than 3 months.

### 2.5 Quality assessment

Revman 5.3 software provided by Cochrane was used to analyze data quality and potential bias. The results were independently assessed by two researchers using the RCT bias risk assessment tool recommended by the Cochrane Handbook and were cross-checked for accuracy. Evaluations included random sequence generation, random concealment, blinding, data integrity, withdrawal, and other biases. The included literature was evaluated for bias risk from seven aspects using decision terms such as “high risk,” “low risk,” and “unclear risk.”

### 2.6 Statistical analysis

Eligible studies were used as sources for data extraction and entered into a standardized spreadsheet. Five outcome measures (eGFR, 24 hpro, Scr, BUN, and ADR) were analyzed. The standardized mean difference was used for continuous variables, whereas relative risk was used for dichotomous variables. Each effect size is expressed as a 95% confidence interval. A traditional meta-analysis using Stata18.0 software was conducted in conjunction with the I^2^ value to quantitatively assess the degree of heterogeneity among the studies. If the I^2^ value exceeded 50%, substantial heterogeneity between the groups was indicated, necessitating the use of a random-effects model. Conversely, if the I^2^ value was less than or equal to 50%, minimal heterogeneity between groups was indicated, which allowed for the selection of a fixed-effects model. In cases with significant heterogeneity, sensitivity, and subgroup analyses were performed to identify potential sources of heterogeneity before deciding whether inclusion should be based on these findings alone. Descriptive analyses were used when the source of heterogeneity could not be determined.

A frequency framework was adopted and a random-effects model was employed for the network meta-analysis. Data preprocessing was performed using the network group command to visualize the network relationships between the intervention measures for each outcome index comparison. Inconsistency tests were conducted when closed loops formed among the interventions to assess the level of agreement between direct and indirect comparisons. Consistency with the study was determined at *p* > 0.05; otherwise, statistical differences were evaluated using the node-splitting method. The surface under the cumulative ranking curve (SUCRA) was used to rank the efficacy indicators of the intervention measures and identify the most effective intervention. Higher SUCRA values indicated better intervention effects. Stata 18.0 software was used to generate a publication bias funnel plot (comparison-correction plot).

## 3 Results

### 3.1 Study characteristics

A PRISMA flowchart of the screening process used in this study is shown in Figure 1. In total, 1,128 articles were retrieved, of which 794 were excluded because of duplications in the Import Endnote software. Additionally, 563 articles did not meet the inclusion criteria and were excluded based on their titles and abstracts. We conducted a thorough review of the full text of the remaining 231 articles with potentially eligible records. Ultimately, this study included 72 articles involving 7,030 patients. The following seven therapeutic interventions were evaluated: placebo, TCM, ACEI, ARB, TCM + ACEI, TCM + ARB, and ACEI + ARB. No statistically significant differences in baseline parameters were observed between the study groups. Table 1 summarizes the clinical and methodological characteristics of each trial along with their main results.

**TABLE 1:** Characteristics of clinical trials and patients.

Study	No of patients		Sex (male %)		Age (years)		Treatment		Duration (months)	GFR (mL/min/1.73 m^2^ )		Serum creatinine (μmol/L)		24hpro (g/d)	
	T	C	T	C	T	C	T	C		T	C	T	C	T	C
Shima et al. (2019)	31	31	58.1	51.6	12.0 ± 3.2	11.9 ± 3.3	ACEI + ARB	ACEI	24	121.3 ± 15.3	120.1 ± 13.8				
Li et al. (2020)	735	735	48	46.3	37.7 ± 10.9	37.1 ± 10.4	TCM	ARB	12	94.2 ± 24.4	95.7 ± 24.0	80.3 ± 25.2	81.8 ± 25.1	1.24 ± 0.67	1.21 ± 0.64
Li et al. (2006)	54	55	24.0	30.9	40 ± 10	41 ± 9	ARB	placebo	36	85.9 ± 37.30	72.47 ± 35.16	114.04 ± 47.74	98.12 ± 42.43	1.80 ± 1.24	2.35 ± 1.71
Zhang et al. (2014)	135	133	72	67	38.1 ± 12.7	37.3 ± 12.5	ARB	TCM	6	106 ± 23	108 ± 24	70.72 ± 19.45	72.49 ± 18.56	1.08 ± 0.45	1.05 ± 0.42
Zhang et al. (2014)	136	133	64	67	37.1 ± 11.1	37.3 ± 12.5	ARB + TCM	TCM	6	106 ± 24	108 ± 24	71.60 ± 20.33	72.49 ± 18.56	1.07 ± 0.44	1.05 ± 0.42
Nakamura et al. (2007)	8	8	62.5	50	34 ± 7	31 ± 8	ARB	ACEI	3			88.4 ± 26.52	97.24 ± 17.68	2.1 ± 0.6	1.9 ± 0.3
Nakamura et al. (2007)	8	8	62.5	50	34 ± 7	31 ± 7	ARB	ARB + ACEI	3			88.4 ± 26.52	97.24 ± 17.68	2.1 ± 0.6	2.0 ± 0.4
Horita et al. (2004)	10	10	50	40	42.7 ± 12	39.6 ± 10.8	ARB	ACEI	6	88.3 ± 62.61	92.5 ± 54.39	77.79 ± 47.53	75.14 ± 58.69	0.81 ± 1.39	0.73 ± 1.14
Horita et al. (2004)	10	11	50	45.5	42.7 ± 12	39.6 ± 10.4	ARB	ARB + ACEI	6	88.3 ± 62.61	91.5 ± 81.59	77.79 ± 47.53	73.37 ± 65.67	0.81 ± 1.39	0.75 ± 0.99
Horita et al. (2006)	16	14	56.3	66.7	42 ± 9	43 ± 10	ARB	ACEI	12	88.0 ± 72.8	89.8 ± 85.68	78.68 ± 46.32	74.26 ± 56.24	0.83 ± 1.76	0.60 ± 0.79
Horita et al. (2006)	16	13	56.3	53.8	42 ± 9	38 ± 9	ARB	ARB + ACEI	12	88.0 ± 72.8	95.3 ± 80.76	78.68 ± 46.32	74.26 ± 63.64	0.83 ± 1.76	0.80 ± 1.19
Renke et al. (2004)	18	18	38.9	66.7	40.4 ± 11.9	43.4 ± 10.1	ARB	ACEI	9					2.17 ± 1.52	2.6 ± 1.69
Renke et al. (2004)	18	16	38.9	68.8	40.4 ± 11.9	37.7 ± 12.7	ARB	ARB + ACEI	9					2.17 ± 1.52	3.25 ± 1.82
Shen et al. (2012)	112	114	51.8	49.1	50.2 ± 10.4	49.1 ± 11.5	ARB	placebo	12	44.8 ± 8.1	44.8 ± 8.5	130.03 ± 23.87	131.72 ± 24.75	1.72 ± 0.47	1.73 ± 0.49
Liang et al. (2017)	26	26	53.85	46.15	38.7 ± 12	37.1 ± 11.7	ARB	ARB + TCM	6	104 ± 25	105 ± 23	73.4 ± 19.5	75.1 ± 22.1	1.09 ± 0.45	1.07 ± 0.43
Zhao (2015)	30	30	50	53.3	32 ± 6.32	32 ± 6.32	ARB	ARB + TCM	3			88.98 ± 12.50	92.46 ± 13.71	1.67 ± 0.25	1.70 ± 0.25
Su and Zhang (2014)	24	24	54.17	50	34.2 ± 8.1	34.4 ± 7.9	ARB	ARB + TCM	3	95.6 ± 5.4	96.2 ± 5.3			1.92 ± 0.66	1.85 ± 0.77
Wang et al. (2014)	78	78	51.28	57.69	39.4 ± 6.2	37.3 ± 5.7	ARB	ARB + TCM	12			102.1 ± 30.3	115.6 ± 27.8	2.47 ± 0.53	2.54 ± 0.41
Xiang et al. (2014)	30	30	60	63.33	51.3 ± 8.2	50.3 ± 9.6	ARB	ARB + TCM	3			92.15 ± 12.03	89.73 ± 12.14	2.09 ± 0.53	1.19 ± 0.45
Duan and Wang (2014)	30	30	36.67	40	33 ± 8	35 ± 9	ARB	ARB + TCM	6					1.35 ± 0.66	1.46 ± 0.70
Xue (2012)	30	30	60	66.67	31.77 ± 10.47	32.87 ± 11.33	ARB	ARB + TCM	3			85.87 ± 20.55	80.70 + 21.11	1.005 ± 0.83	1.055 ± 0.86
Li et al. (2012)	30	30	56.67	53.33	34.5 ± 11.7	32.8 ± 12.7	ARB	ARB + TCM	3			86.4 ± 25.4	87.4 ± 26.6	1.35 ± 0.63	1.48 ± 0.51
Guo et al. (2010)	35	35	52.29	62.86	28.86 ± 8.95	30.60 ± 8.96	ARB	TCM	12			91.41 ± 11.67	89.59 ± 8.57	1.44 ± 0.27	1.52 ± 0.35
Zhou and Yao (2010)	30	30	56.67	53.33	32.8 ± 12.6	33.2 ± 11.8	ACEI	TCM	12			85.87 ± 42.96	86.66 ± 43.3	2.40 ± 1.12	2.45 ± 1.01
Wang (2020a)	17	17	58.82	64.71	43.75 ± 3.92	42.61 ± 4.22	ARB	ARB + TCM	6			138.08 ± 13.51	137.15 ± 12.66	1.73 ± 0.60	1.71 ± 0.45
Wei (2019)	35	35	60	54.29	37.65 ± 5.58	39.57 ± 5.16	ARB	ARB + TCM	3			108.36 ± 7.27	108.91 ± 7.36		
Cai (2018)	34	34	52.94	55.88	46.12 ± 9.05	45.78 ± 8.83	ARB	ARB + TCM	6	105.12 ± 12.93	104.29 ± 13.06	136.36 ± 14.34	134.21 ± 14.34	1.68 ± 0.48	1.70 ± 0.51
Xu (2020)	29	29	58.62	55.17	40.03 ± 2.49	39.65 ± 2.81	ACEI	ACEI + TCM	3			110.20 ± 8.05	109.59 ± 7.63	1.68 ± 0.69	1.71 ± 0.64
Hou et al. (2009)	30	30	60	60	34.5 ± 13.4	38.6 ± 14.2	ACEI	ACEI + ARB	3					2.2 ± 0.6	2.2 ± 0.6
Liu et al. (2007b)	20	20	70	65	13–58	14–61	ARB	ACEI	6			126.23 ± 74.24	126.78 ± 72.04	2.21 ± 1.43	2.18 ± 1.36
Liu et al. (2007b)	20	21	70	76.19	13–58	13–54	ARB	ACEI + ARB	6			126.23 ± 74.24	126.06 ± 73.14	2.21 ± 1.43	2.16 ± 1.42
Chang et al. (2021a)	29	29	44.83	48.28	39.03 ± 9.14	37.34 ± 6.77	ARB	TCM	4			90.66 ± 28.5	87.88 ± 23.12	1.75 ± 0.54	1.6 ± 0.46
Wang (2021)	27	25	48.19	52	39.96 ± 9.29	37.84 ± 6.55	ARB	TCM	6			93.29 ± 26.37	96.2 ± 25.91	1.68 ± 0.46	1.51 ± 0.49
Long (2021)	30	30	43.3	36.67	40.40 ± 12.73	41.87 ± 13.11	ACEI	ACEI + TCM	3			67.87 ± 9.36	65.40 ± 9.26	1.50. ± 0.22	1.50 ± 0.29
Lu and Ma (2021)	30	30	46.67	50	41.00 ± 11.69	42.27 ± 1 1.46	ARB	ACEI + ARB	3			104.29 ± 40.1	106.8 ± 42.85	1.91 ± 0.92	1.85 ± 1.17
Guo and Du. (2023)	50	50	54	52	41.16 ± 3.33	42.06 ± 3.84	ARB	ARB + TCM	6			82.32 ± 4.51	81.65 ± 3.65		
Guan et al. (2005)	30	32	53.3	53.13	30.4 ± 6.2	31.2 ± 6.8	TCM	ARB + TCM	3			212.8 ± 60.4	216.4 ± 56.2	2.24 ± 1.32	2.26 ± 1.27
Wang et al. (2010a)	32	32	53.13	53.13	31.30 ± 9.75	31.10 ± 9.83	ACEI	TCM	3			88.5 ± 20.8	85.2 ± 29.0	1.47 ± 0.82	1.48 ± 0.84
Han and Qiu (2010)	26	29	53.85	51.72	38 ± 16.3	37 ± 17.4	ACEI	ACEI + TCM	3			95.5 ± 7.6	98.7 ± 9.7	1.93 ± 0.93	1.89 ± 0.58
Huang et al. (2008b)	35	35	24	22	28.23 ± 10.32	27.63 ± 15.55	ACEI	TCM	6					0.83 ± 0.56	0.86 ± 0.42
Liu et al. (2010)	29	32	58.62	65.63	32.72 ± 8.76	31.40 ± 8.74	ACEI	TCM	4					1.372 ± 0.372	1.324 ± 0.396
Liu et al. (2007a)	32	32	53.13	53.13	36.0 ± 12.0	36.0 ± 12.0	ACEI	ACEI + TCM	6			123.3 ± 46.4	121.1 ± 47.2	2.1 ± 1.4	2.0 ± 1.4
Gao (2009)	29	30	48.28	60	NA	NA	ACEI	ACEI + TCM				75.02 ± 18.22	74.44 ± 19.76	1.44 ± 0.55	1.34 ± 0.46
Zhang et al. (2019a)	40	40	NA	NA	NA	NA	ARB	TCM	6			110.98 ± 35.93	113.95 ± 39.60	1.19 ± 0.53	1.34 ± 0.75
Pan et al. (2017)	31	31	61.29	54.39	31.4 ± 12.3	33.8 ± 1.9	ARB	ARB + TCM	3			88.23 ± 35.34	86.57 ± 32.89	1.38 ± 0.46	1.34 ± 0.56
Huang and He (2011)	17	33	58.82	54.55	NA	NA	ACEI	ACEI + TCM	3			135.78 ± 49.15	136.12 ± 50.57	2.71 ± 0.42	2.69 ± 0.47
Luo et al. (2023)	40	40	45	35	48. 95 ± 7. 26	47. 84 ± 6.35	ACEI	ACEI + TCM	3			128.39 ± 5.62	132.41 ± 6.55	2.39 ± 0.62	2.41 ± 0.5
Wei et al. (2017)	30	30	63.33	56.67	36.31 ± 8.96	36.26 ± 8.91	ACEI	TCM	3			67.68 ± 11.46	63.53 ± 10.44	0.71 ± 0.43	0.72 ± 0.45
Chen et al. (2022)	30	30	56.67	53.33	37.5 ± 10.64	38.27 ± 10.72	ARB	ARB + TCM	3			70.56 ± 11.21	71.37 ± 11.02	2.13 ± 0.31	2.05 ± 0.32
Xu et al. (2009)	25	27	44	48.15	39.2 ± 12.39	36.8 ± 12.17	ACEI	TCM	6			91.56 ± 30.68	83.41 ± 22.37	1.12 ± 0.44	1.32 ± 0.50
Huang (2008a)	30	30	60	56.67	NA	NA	ACEI	ACEI + TCM	3			74.23 ± 19.29	76.07 ± 20.36	1.39 ± 0.67	1.29 ± 0.57
Wang et al. (2021c)	45	45	57.78	55.56	52.43 ± 13.11	54.38 ± 11.25	ARB	ARB + TCM	6			78.78 ± 19.95	77.24 ± 20.32	2.11 ± 0.49	2.16 ± 0.58
Wang et al. (2020d)	35	35	51.43	57.14	53.48 ± 11.75	51.63 ± 12.51	ARB	ARB + TCM	6			79.34 ± 20.93	81.03 ± 20.62	2.14 ± 0.56	2.24 ± 0.61
Gan et al. (2019)	39	39	51.28	60	32.95 ± 3. 26	33.27 ± 3. 31	ARB	ARB + TCM	3					2.90 ± 0.28	2.88 ± 0.27
Han et al. (2016)	50	50	40	44	34.36 ± 8.05	35.28 ± 8.24	ACEI	ACEI + TCM	6			92.47 ± 17.38	90.25 ± 18.65	2.15 ± 0.77	2.03 ± 0.75
Zhao et al. (2021a)	40	40	57.5	55	37.9 ± 11.6	35.8 ± 10.8	ARB	ARB + TCM	6			93.36 ± 12.74	95.90 ± 10.30	1.31 ± 0.11	1.34 ± 0.25
Chen et al. (2007)	66	65	60.60	56.92	33.65 ± 12.36	33.98 ± 11.05	ACEI	TCM	4			87.19 ± 40.21	79.42 ± 31.29	1.40 ± 0.98	1.32 ± 0.87
Chen et al. (2006)	34	36	38.24	50	30.97 ± 8.67	30.33 ± 12.26	ACEI	TCM	4			68.47 ± 19.41	71.80 ± 35.74	1.17 ± 0.76	1.22 ± 0.86
Xiang et al. (2009)	32	32	53.13	56.25	37.26 ± 19.66	32.56 ± 13.16	ACEI	ACEI + TCM	3			142.08 ± 30.98	139.86 ± 33.27	1.91 ± 0.97	1.87 ± 1.05
Huang et al. (2012)	25	25	NA	NA	NA	NA	ACEI	ACEI + TCM	3			72.67 ± 13.45	72.67 ± 13.45	2.03 ± 0.67	2.21 ± 1.01
Wang and Shou (2010b)	28	32	NA	NA	NA	NA	ACEI	ACEI + ARB	6					2.3 ± 0.5	2.3 ± 0.5
Li et al. (2018)	38	39	34.21	35.90	42. 20 ± 10. 89	42. 08 ± 9. 71	ACEI	ACEI + TCM	3			90.94 ± 33.72	90.88 ± 34.81	1.15 ± 0.74	1.23 ± 0.77
Yang and Chen (2010)	21	21	NA	NA	NA	NA	ACEI	ACEI + TCM	3			99.45 ± 37.83	98.26 ± 38.72	1.48 ± 0.66	1.51 ± 0.65
Pan et al. (2022)	60	60	32	34	32.6 ± 6.9	30.6 ± 8.9	ARB	TCM	6			74.08 ± 16.08	75.33 ± 15.79	1.23 ± 0.33	1.18 ± 0.30
Meng et al. (2015)	43	44	23	24	38.74 ± 11.67	40.56 ± 11.71	ACEI	TCM	12			91.46 ± 34.28	92.34 ± 36.22	1.99 ± 0.51	2.08 ± 0.42
Han (2012)	26	26	34.62	42.31	NA	NA	ARB	ARB + TCM	3			95.8 ± 136	98.7 ± 143.4	1.68 ± 1.18	1.57 ± 0.94
Zhang et al. (2016)	30	30	66.67	70	32. 00 ± 6. 06	33. 00 ± 9. 53	ACEI	TCM	18			86.14 ± 18.69	88.02 ± 17.55	0.74 ± 0.18	0.78 ± 0.14
Wang et al. (2018)	25	25	52	56	32.3 ± 3.9	31.9 ± 3.6	ARB	ARB + TCM	3	68.67 ± 18.74	67.41 ± 19.54			2.33 ± 0.43	2.31 ± 0.41
Zhao et al. (2022)	48	49	54.17	51.02	32.75 ± 3.08	33.03 ± 2.77	ARB	ARB + TCM	3	61.74 ± 6.22	61.83 ± 6.14	95.48 ± 9.26	95.62 ± 9.15	1.78 ± 0.25	1.70 ± 0.29
Wang et al. (2020c)	39	40	43.59	37.5	43. 82 ± 12. 65	43. 55 ± 12. 35	ARB	TCM	6	82.09 ± 26.07	81.00 ± 25.18	91.56 ± 35.42	91.13 ± 32.88	1.36 ± 0.67	1.49 ± 0.63
Zhang et al. (2019b)	20	20	55	45	43. 40 ± 11. 31	45. 95 ± 10. 35	ARB	TCM	6	72. 55 ± 29. 43	67. 80 ± 26. 85	110.98 ± 35.93	113.95 ± 39.60		
Yao and Zhou (2011)	32	32	56.25	53.13	NA	NA	ACEI	ACEI + TCM	3			88.5 ± 20.8	85.2 ± 29.0	1.47 ± 0.82	1.48 ± 0.84
Wang et al. (2020b)	61	61	47.54	50.82	41.55 ± 14.17	42.54 ± 14.28	ARB	ARB + TCM	6	68.89 ± 3.59	64.84 ± 3.54	116.67 ± 6.23	119.55 ± 6.82	0.99 ± 0.10	1.13 ± 0.12
Wang et al. (2019)	78	78	47.43	51.28	42.71 ± 10.41	42.67 ± 10.47	ARB	ARB + TCM	6	69.77 ± 3.04	67.37 ± 2.81	116.60 ± 5.25	116.79 ± 5.14	1.10 ± 0.09	1.23 ± 0.09
Guo (2019)	30	30	50	46.67	35.93 ± 12.99	35.50 ± 11.73	ARB	ARB + TCM	3			69.33 ± 14.28	75.53 ± 16.57	1.50 ± 0.76	1.66 ± 0.76
Li et al. (2023)	35	38	54.26	55.26	37.18 ± 7.24	36.42 ± 8.89	ARB	ARB + TCM	3			88.84 ± 5.52	89.21 ± 4.47	3.78 ± 0.58	3.74 ± 0.53
Jia (2012)	33	33	NA	NA	NA	NA	ACEI	ACEI + TCM	3			85.86 ± 5.46	85.05 ± 3.18	1.92 ± 0.95	1.81 ± 1.04
Duan (2011)	30	30	23.33	33.33	NA	NA	ACEI	ACEI + TCM	3					1.44 ± 0.73	1.49 ± 0.77

T, treatment; C, control; ACEI, angiotensin-converting enzyme inhibitor; TCM, traditional Chinese medicine; ARB, angiotensin II, receptor blocker; GFR, glomerular filtration rate; 24hpro, 24-h urinary protein.

### 3.2 Assessment of risk of bias

**FIGURE 2:**
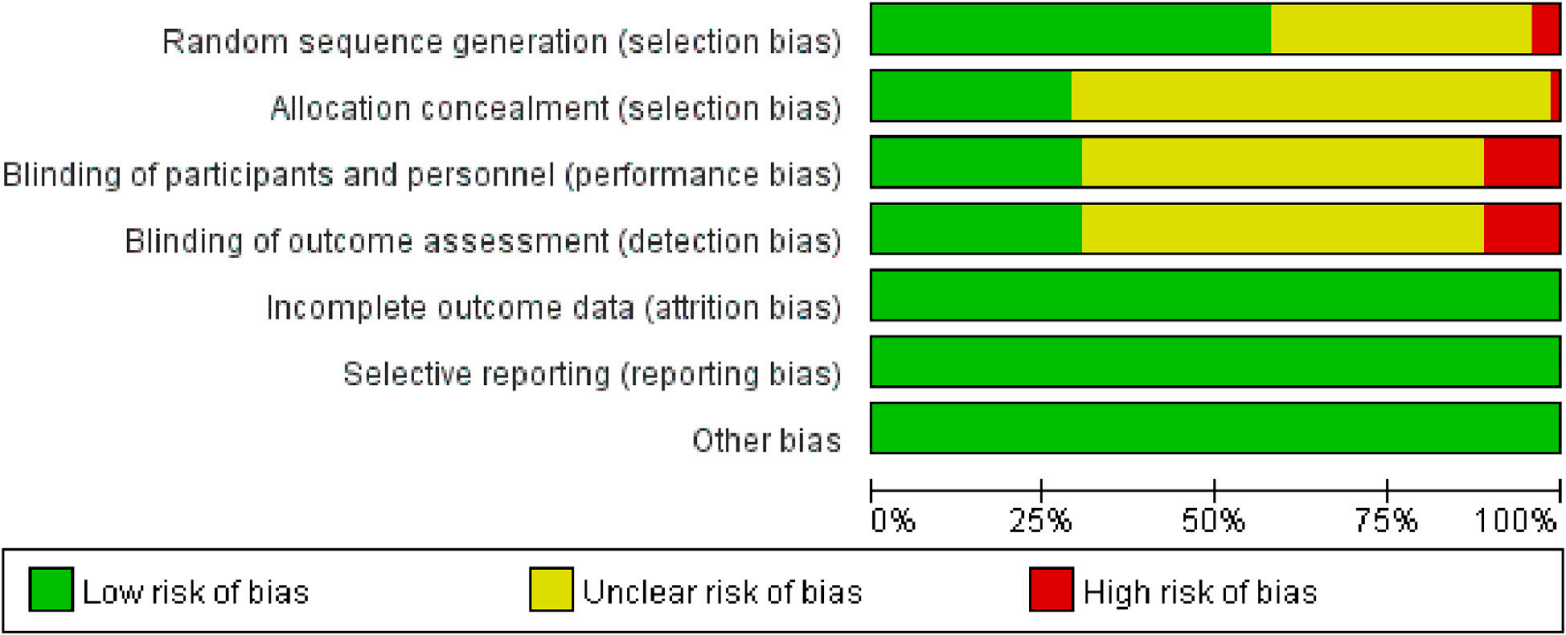
Risk of bias graph.

The 72 studies were assessed for risk of bias. Explicit random sequence generation was reported in 43 studies, whereas randomization was mentioned without describing the method in 26. The randomization procedures in three additional studies were inadequately implemented. Specific implementation measures for allocation concealment are mentioned in the literature. Eighteen studies mentioned specific blinding practices; however, only seven of these studies achieved the ideal implementation of blinding. Complete outcome data were available for all included studies. The results of the risk-assessment bias analysis are shown in Figure 2.

### 3.3 Inconsistency test

The selected treatments were subjected to a node-split analysis, which revealed no statistically significant differences (all *p* > 0.05). This analysis demonstrated the absence of disparities between direct and indirect evidence (Supplementary Table S2). The results of the heterogeneity tests among the multiple interventions are presented in Supplementary Table S3.

### 3.4 Outcome measures

#### 3.4.1 eGFR

**FIGURE 3:**
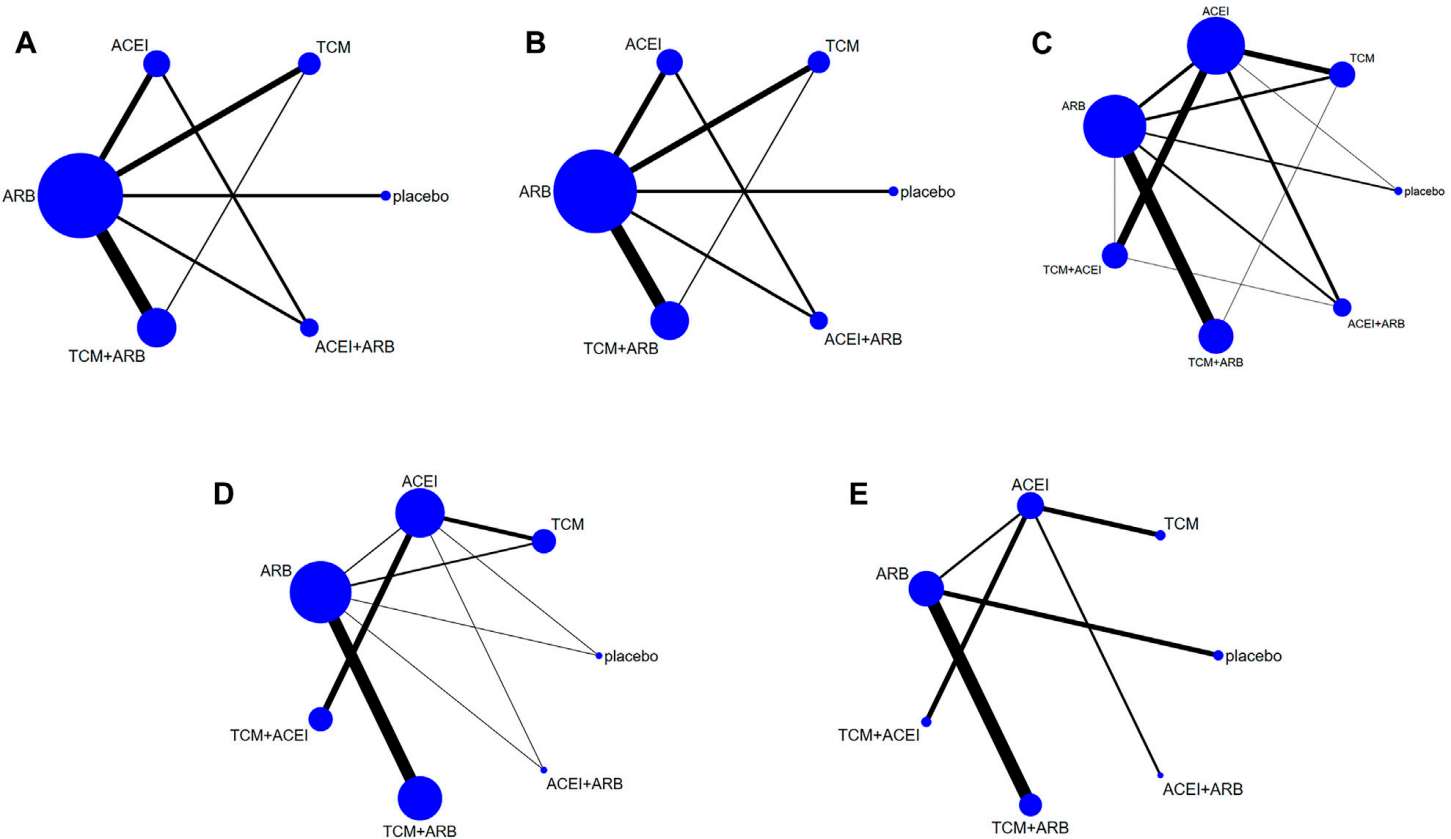
Observation index network for the outcomes **(A)** estimated glomerular filtration rate, **(B)** serum creatinine, **(C)** 24-h urinary protein, **(D)** blood urea nitrogen, and **(E)** adverse drug reactions. TCM, traditional Chinese medicine; ACEI, angiotensin-converting enzyme inhibitor; ARB, angiotensin receptor blocker.

**FIGURE 4:**
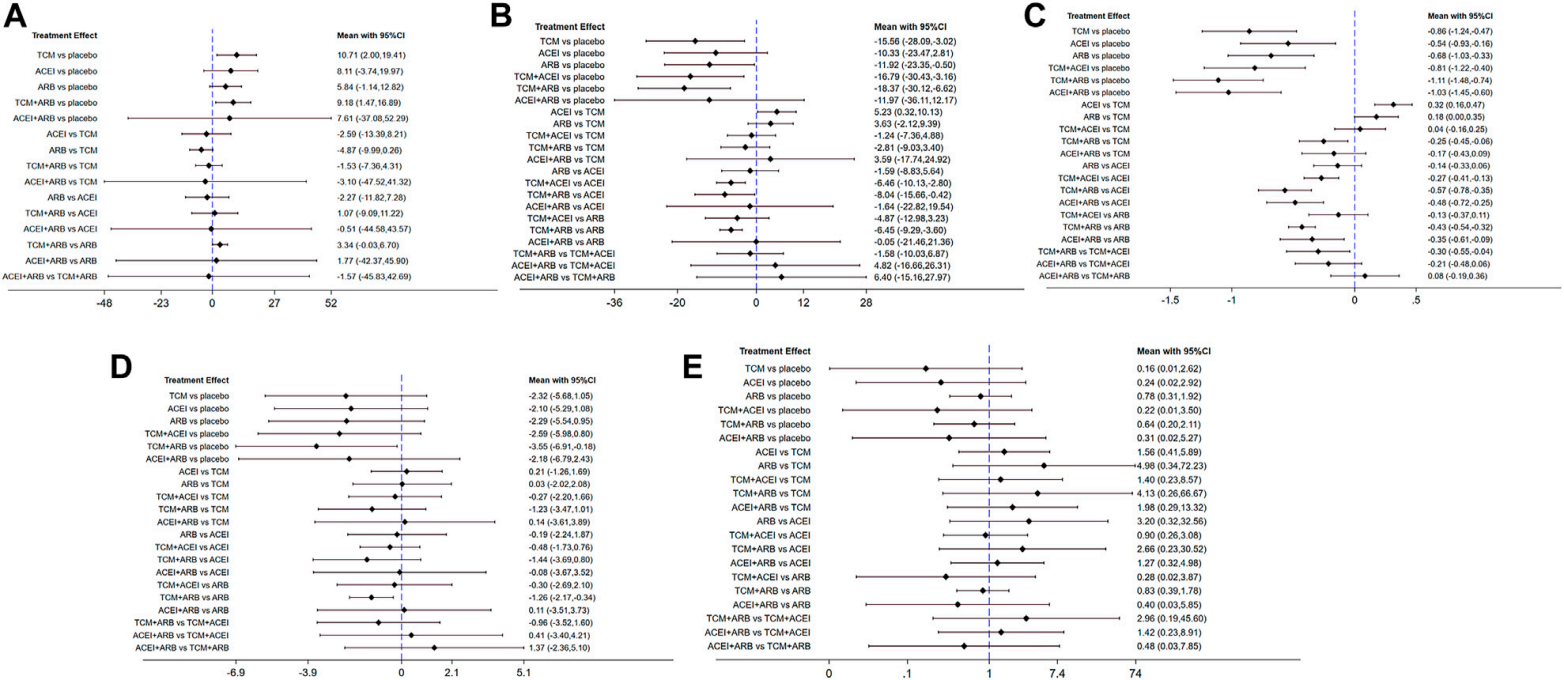
Forest plots for prediction through network meta-analysis using observational indices for the outcomes **(A)** estimated glomerular filtration rate, **(B)** serum creatinine, **(C)** 24-h urinary protein, **(D)** blood urea nitrogen, and **(E)** adverse drug reactions. TCM, traditional Chinese medicine; ACEI, angiotensin-converting enzyme inhibitor; ARB, angiotensin receptor blocker.

**FIGURE 5:**
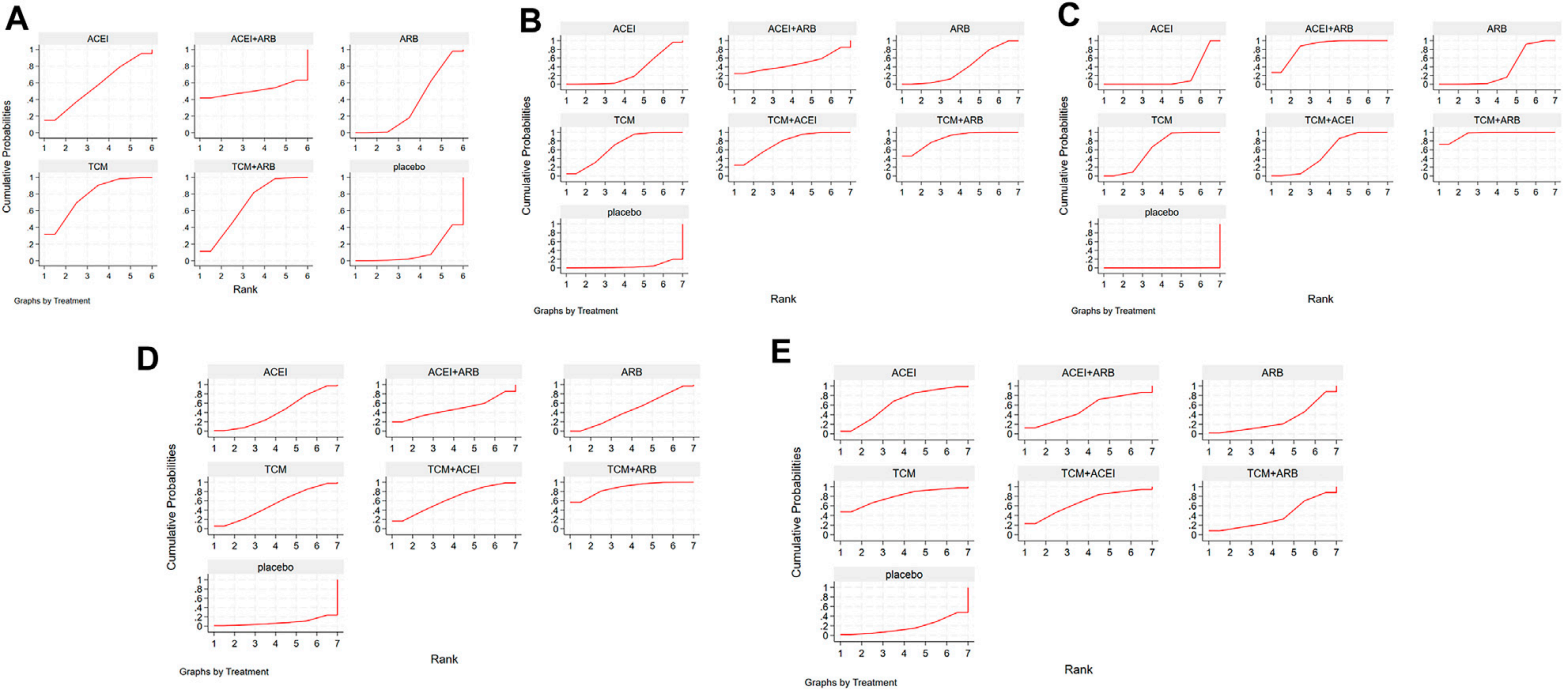
Surface under the cumulative ranking curves depicting the impact of diverse clinical interventions on various indicators: **(A)** estimated glomerular filtration rate, **(B)** serum creatinine, **(C)** 24-h urinary protein, **(D)** blood urea nitrogen, and **(E)** adverse drug reactions. TCM, traditional Chinese medicine; ACEI, angiotensin-converting enzyme inhibitor; ARB, angiotensin receptor blocker.

**FIGURE 6:**
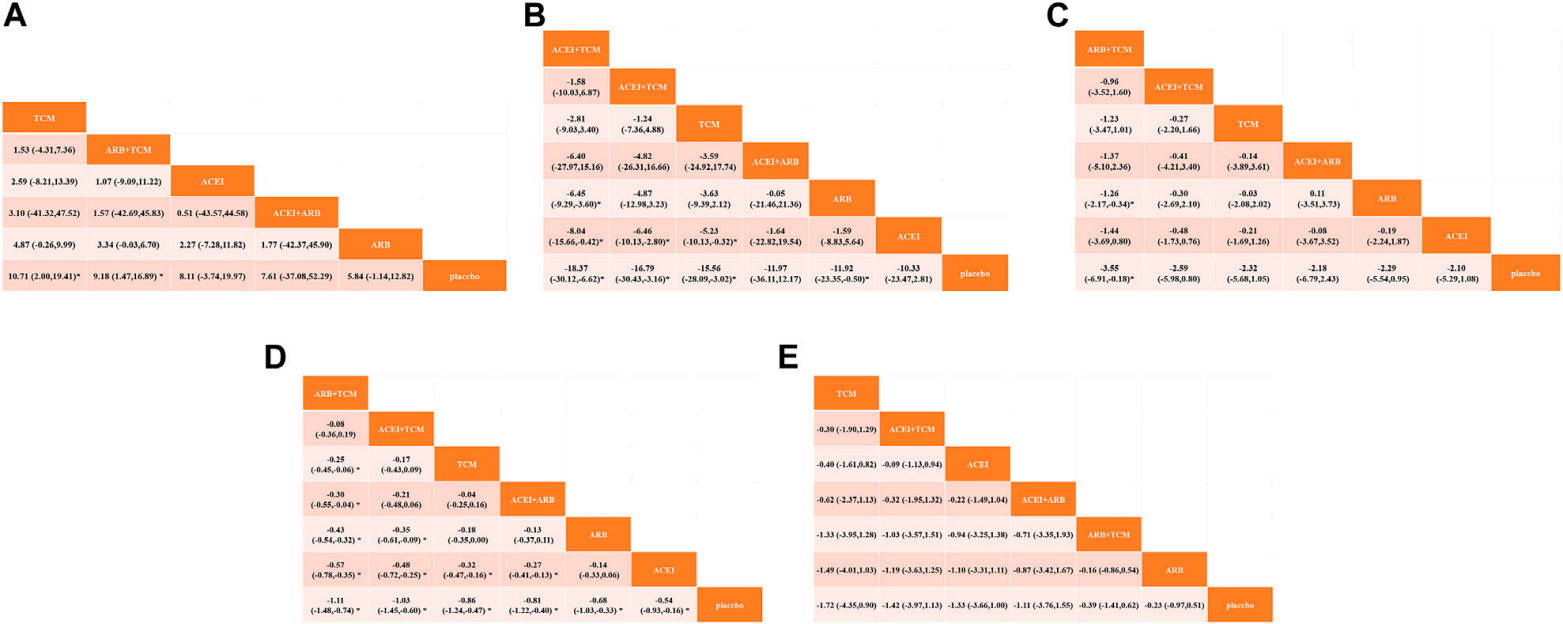
Comparative analysis of therapeutic interventions on diverse clinical indicators: **(A)** estimated glomerular filtration rate, **(B)** serum creatinine, **(C)** 24-h urinary protein, **(D)** blood urea nitrogen, and **(E)** adverse drug reactions. TCM, traditional Chinese medicine; ACEI, angiotensin-converting enzyme inhibitor; ARB, angiotensin receptor blocker.

**FIGURE 7:**
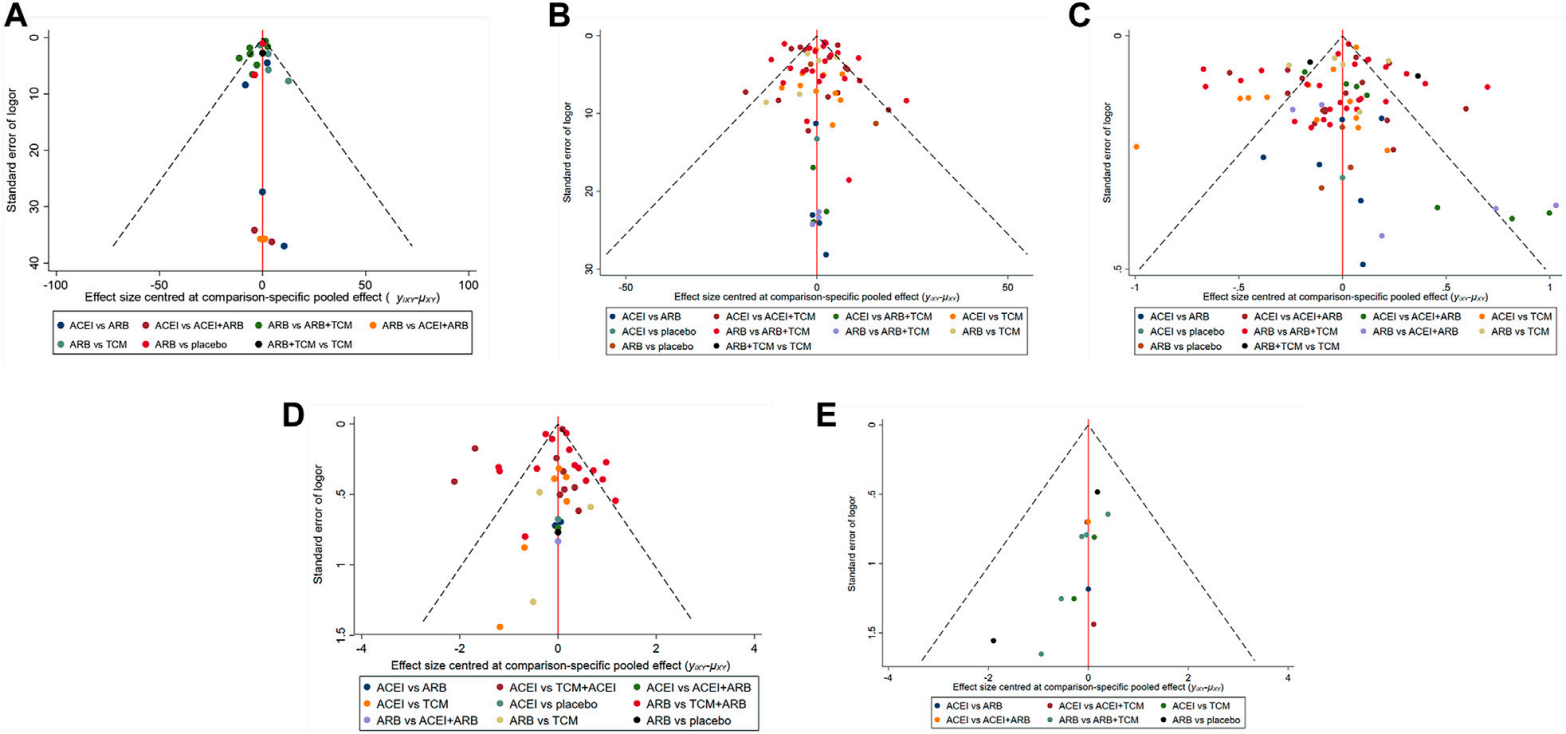
Funnel plot illustrating the treatment effects of diverse clinical interventions on various indicators: **(A)** estimated glomerular filtration rate, **(B)** serum creatinine, **(C)** 24-h urinary protein, **(D)** blood urea nitrogen, and **(E)** adverse drug reactions. TCM, traditional Chinese medicine; ACEI, angiotensin-converting enzyme inhibitor; ARB, angiotensin receptor blocker.

Seventeen studies involving 2,974 patients with changes in eGFR from baseline were included in the analysis (Figure 3A). The network graph revealed that most comparisons were made between the ARB and TCM + ARB groups. Subsequently, we conducted 15 pairwise comparisons using network meta-analyses, two of which showed statistically significant results (Figure 4A). Forest plots generated using Stata software, which indicate the prediction intervals, are presented in Figure 5A. In our analysis, placebo was used as the control group. The eGFRs of the patients were improved by TCM, ACEI, ARB, TCM + ARB, and ACEI + ARB compared with those of the control group. According to the SUCRA ranking analysis for improving eGFR levels (Figure 6A), TCM intervention was found to be the most effective, followed by TCM + ARB, ACEI, ACEI + ARB, ARB, and placebo (Supplementary Table S4). The publication bias assessment is presented in Figure 7A, where each dot represents an included study and different colors indicate different interventions. The comparatively corrected funnel plot suggested acceptable symmetry but also indicated the possibility of publication bias.

#### 3.4.2 Scr

A total of 57 studies involving 4,365 patients with changes in Scr levels from baseline were included (Figure 3B). The network graph revealed that most comparisons were between the ARB and TCM + ARB groups. We conducted a network meta-analysis of Scr reduction based on the included studies, resulting in 21 paired comparisons, of which eight showed statistical significance (Figure 4B). Forest plots displaying the prediction intervals are presented in Figure 5B, with the placebo intervention serving as a control. Compared with the control group, the TCM, ACEI, ARB, TCM + ARB, and ACEI + ARB interventions demonstrated a reduction in the patients’ Scr levels. According to the SUCRA ranking analysis (Figure 6B), TCM + ARB intervention was the most effective approach for reducing Scr levels. The order of probability size was as follows: TCM + ARB > TCM + ACEI > ACEI + ARB > ARB > ACEI > placebo (Supplementary Table S4). The results of the publication bias assessment are shown in Figure 7B. Each dot represents an included study, with different colors indicating different interventions. The comparatively corrected funnel plot suggested acceptable symmetry but also indicated the possibility of publication bias.

#### 3.4.3 24hpro

A total of 68 studies involving 6,520 patients with changes at 24 hpro from baseline were included (Figure 3C). The network graph revealed that the majority of comparisons were made between the ARB and TCM + ARB groups. We conducted a network meta-analysis of the included studies to assess their efficacy in reducing 24 hpro, resulting in 21 paired comparisons, of which eight showed statistical significance (Figure 4C). The generated forest plots are presented in Figure 5C, where the placebo intervention was used as the control group. Compared to the control group, the TCM, ACEI, ARB, TCM + ARB, and ACEI + ARB groups demonstrated a significant reduction in 24 hpro levels. According to the SUCRA ranking analysis, TCM + ARB intervention was the most effective approach for reducing 24 hpro levels (Figure 6C). The order of probability size was as follows: TCM + ARB > ACEI + ARB > TCM > ACEI + TCM > ARB > ACEI > placebo (Supplementary Table S4). The results of the publication bias assessment are shown in Figure 7C. Each dot represents an included study, and different colors indicate different interventions. The comparative corrected funnel plot indicated acceptable symmetry but also suggested the possibility of publication bias.

#### 3.4.4 BUN

A total of 36 studies involving 2,358 patients with a change in BUN from baseline (Figure 3D) were included. The network graph revealed that most comparisons were made between the ARB and TCM + ARB groups. We conducted a network meta-analysis of the included studies to assess the reduction in BUN levels, which resulted in 21 paired comparisons, two of which showed statistical significance (Figure 4D). Forest plots are presented in Figure 5D, with the placebo intervention serving as the control group. Compared to the control, TCM, ACEI, ARB, TCM + ARB, and ACEI + ARB demonstrated efficacy in reducing BUN levels. According to the SUCRA ranking analysis (Figure 6D), the TCM + ARB intervention was the most effective approach for BUN reduction among all the interventions considered. The order of probability size was as follows: TCM + ARB > TCM + ACEI > TCM > ACEI + ARB > ARB > ACEI > placebo (Supplementary Table S4). Publication bias assessment results are displayed in Figure 7D, where each dot represents an included study, and different colors indicate different interventions. The comparatively corrected funnel plot suggested acceptable symmetry but also indicated the possibility of publication bias.

#### 3.4.5 ADR

Thirteen studies were included, with 88 adverse events reported in 1,158 patients (Figure 3E). The network graph illustrates the predominant comparisons between the ARB and TCM + ARB groups. We conducted a network meta-analysis of the included studies on ADRs, comprising 21 paired comparisons; however, none of these comparisons yielded statistically significant results (Figure 4E), suggesting that there may be no difference in terms of ADR causation among the interventions. Forest plots displaying the prediction intervals are shown in Figure 5E, with the placebo intervention serving as a control. Compared with the control, TCM, ACEI, ARB, TCM + ARB, and ACEI + ARB interventions demonstrated reductions in patient ADRs. According to the SUCRA rankings for ADR reduction (Figure 6E), the TCM intervention appeared to be potentially superior among all interventions evaluated. The order of probability size was as follows: TCM > TCM + ACEI > ACEI > ACEI + ARB > ARB + TCM > ARB > placebo (Supplementary Table S4). Publication bias analysis results are presented in Figure 7A, where each dot represents an included study and different colors indicate different interventions. The comparatively corrected funnel plot indicated acceptable symmetry but also suggested potential publication bias.

## 4 Discussion

In this network meta-analysis, we conducted a comprehensive evaluation of the effects of ACEIs, ARBs, TCM, and combination treatment strategies on key renal outcomes in patients with IgAN. Our findings demonstrate that the addition of TCM significantly enhances the efficacy of ACEI or ARB therapy compared with ACEI or ARB alone, as well as with placebo. Specifically, the combined use of TCM with ACEI or ARB effectively reduced 24 hpro, Scr, and BUN levels. Moreover, our results suggest that combining an ACEI with TCM is more effective than using an ACEI alone for preserving renal function and that combining an ARB with TCM is more effective than using an ARB alone. No statistically significant differences in ADRs were observed among the studies.

ACEIs and ARBs are recommended by the KDIGO for the initial treatment of IgAN, as they exhibit a favorable renal protective effect, which has been substantiated in some preliminary studies (Coppo, 2019; Rovin et al., 2021; Floege et al., 2022). However, robust evidence comparing the efficacies of ACEIs and ARBs is lacking. The efficacy of RAS-blockers is often suboptimal in patients with hyperkalemia and severe renal dysfunction. Moreover, these drugs are often associated with adverse effects including dry cough, edema, and fatigue (Whitlock et al., 2023; Yao et al., 2023).

TCM and its decoctions have been extensively used in China to treat IgAN and its associated complications, resulting in a vast accumulation of clinical experience. In recent years, TCM has been extensively used to manage IgAN and its associated complications. Moreover, an increasing number of reports have highlighted the efficacy of TCM combined with ACEIs/ARBs for the treatment of IgAN. Numerous studies have demonstrated that this combination therapy exerts a significant therapeutic effect on IgAN by controlling disease progression and improving clinical symptoms, with minimal side effects and high safety (Li et al., 2020; Wang et al., 2021b; Wang et al., 2022; Pan et al., 2023). The findings of our study also demonstrated that the combination of TCM with ACEI/ARB exhibited superior efficacy compared to ACEI/ARB monotherapy in terms of reducing 24 hpro, eGFR, Scr, and BUN levels and ultimately delayed kidney disease progression.

Mechanistic studies have suggested that TCM exerts its therapeutic effects on IgAN via multiple targets and pathways (Ma et al., 2023). Importantly, extensive and in-depth data mining regarding the application of TCM in treating IgAN facilitates the acquisition of ancient clinical experiences, enhances the understanding of the theoretical and practical aspects of TCM for IgAN treatment, and enriches the therapeutic options for managing this condition. Our findings provide evidence supporting the efficacy and safety of current clinical treatments for IgAN to a certain extent, thereby offering a new and comprehensive framework for promoting clinical practice guidelines. This contributes to expanding the range of therapeutic options available for the management of IgAN.

Common ADRs associated with ACEIs include cough, dizziness, and headache. However, no instances of liver damage have been reported. Common adverse effects of ARBs include hyperkalemia, abdominal distension, and dizziness. The adverse effects observed with ACEIs and ARBs were similar to those observed with ACEIs or ARBs alone. TCM commonly elicits abdominal distension and diarrhea as its main ADR. Mild liver damage and menstrual disturbances have been predominantly observed in studies involving *Tripterygium wilfordii* and are linked to gonadal impairment (Fu et al., 2012; Chang et al., 2021b). It is worth mentioning that the differences in the incidence of ADRs among the various interventions were not statistically significant. Nonetheless, it is advisable to regularly monitor hepatic function, sperm motility, and the menstrual cycle in patients undergoing treatment with TCM *Tripterygium wilfordii*. Furthermore, prudent consideration should be given to the appropriate administration of hepatoprotective agents.

The strengths of this study lie primarily in the implementation of a systematic search method to minimize the potential impact of publication bias, the rationality of the analysis, and the reliability of the obtained results. Network meta-analysis was employed to construct a comprehensive network that incorporated both direct and indirect comparisons, enabling a more thorough comparative analysis of various interventions for IgAN treatment. Sensitivity analyses demonstrated minimal changes in primary outcome measures, thus enhancing the overall credibility of these assessments.

Unfortunately, the limited use of TCM outside China has led us and other researchers to include studies from the same population, which introduces the possibility of publication bias. In our study, there were also insufficient data on patients using different treatment regimens, owing to the inclusion of patients at various stages of IgAN. Therefore, we assessed the efficacy of the major drug classes in the treatment of IgAN. Subgroup analyses involving drugs, different dosages, and varying stages of IgAN failed to clarify the effectiveness of diverse treatment regimens for different stages of IgAN. Among the RCTs included in our analysis, patients exhibited varying degrees of severity at 24 hpro, eGFR, Scr, and BUN levels, along with other indicators, resulting in inconsistent treatment outcomes that may contribute to heterogeneity and impact the study results. Additionally, some studies were deemed to be of low quality as they did not explicitly describe whether allocation concealment or blinding occurred; this lack of information could potentially influence the analysis outcomes.

In the heterogeneity correlation test, heterogeneity among several interventions was not statistically significant and was often below 50%, which is considered within an acceptable range. Only a few studies on TCM interventions have shown a heterogeneity of >50%. We attribute this to the substantial differences in the various interventions included in TCM, variations in the composition of the TCM decoction, and discrepancies in patient indications, such as qi and yin deficiency, qi stagnation, and blood stasis. In summary, it is crucial to conduct high-quality, multicenter, large-sample, double-blind RCTs with long-term follow-up to enhance the robustness of our findings.

## 5 Conclusion

This network meta-analysis integrated multiple clinical datasets and connected multiple RCTs to achieve an indirect comparison of seven different interventions for the treatment of IgAN, effectively addressing the issue of disparate efficacy and priority resulting from a lack of direct comparisons. The results demonstrated that the combination of ACEI + TCM and ARB + TCM, along with TCM treatment alone, significantly decreased the 24 hpro, eGFR, Scr, and BUN levels in patients with IgAN. Among these, the combination of ARBs and TCM resulted in the most favorable outcomes. Combining TCM with conventional therapy for patients with IgAN enhances its protective effects and superiority without increasing the occurrence of ADRs. The pathogenesis of IgAN, which is one of the most prevalent primary glomerular diseases in clinical practice, is complex. Thus, the treatment challenge lies in the heterogeneity of its clinical manifestations and prognosis, necessitating individualized treatment plans (El Karoui et al., 2024). Therefore, combination TCM therapies can be used to enhance therapeutic outcomes based on individual patient circumstances. The severity of symptoms and signs must be considered when devising individualized treatment plans while simultaneously emphasizing the advantages of comprehensive TCM treatments.

## Data Availability

The original contributions presented in the study are included in the article/Supplementary Material, further inquiries can be directed to the corresponding authors.

## Abbreviations

24 hpro, 24-h urinary protein; ACEI, angiotensin-converting enzyme inhibitor; ADR, adverse drug reaction; ARB, angiotensin receptor blocker; BUN, blood urea nitrogen; eGFR, estimated glomerular filtration rate; IgAN, immunoglobulin A nephropathy; KDIGO, Kidney Disease: Improving Global Outcomes; RAS, renin-angiotensin system; PRISMA, Preferred Reporting Items for Systematic Reviews and Meta-Analyses; PROSPERO, International Registry of Prospective Systematic Reviews; RCT, randomized controlled trial; Scr, serum creatinine; SUCRA, surface under the cumulative ranking curve; TCM, traditional Chinese medicine.
