# Supplementary material for: Efficacy of traditional Chinese medicine versus angiotensin-converting enzyme inhibitors, angiotensin receptor blockers, and their combinations in the treatment of IgA nephropathy: a systematic review and network meta-analysis
Source: Front Pharmacol. 2024 Mar 21;15:1374377. doi: 10.3389/fphar.2024.1374377 (PMC10991836; doi:10.3389/fphar.2024.1374377)
Supplement: Supplementary file 1 [file Table1.DOCX]

Supplementary Material

**Supplementary Table S1 Search strategy for five electronic databases**

| Database | Search strategy | No. |
| --- | --- | --- |
| PubMed | #1: ((((Glomerulonephritides, IgA[MeSH Terms]) OR (Berger's Disease[MeSH Terms])) OR (nephropathy, IgA[MeSH Terms])) OR (IgA Glomerulonephritis[MeSH Terms])) OR (IgA Nephropathy[MeSH Terms])  #2: (((IgA nephropathy[Title/Abstract]) OR (immunoglobulin a nephropathy[Title/Abstract])) OR (immunoglobulin a nephritis[Title/Abstract])) OR (IgA nephritis[Title/Abstract])  #3: #1 OR #2  #4: ((Traditional Chinese Medicine[MeSH Terms]) OR (Chinese Traditional Medicine[MeSH Terms])) OR (drug therapy[MeSH Terms])  #5: (((((((Traditional Chinese Medicine[Title/Abstract]) OR (Chinese Herbal Drugs[Title/Abstract])) OR (Chinese Plant Extracts Oriental Medicine[Title/Abstract])) OR (Medicinal Plant[Title/Abstract])) OR (Pharmaceutical Plant[Title/Abstract])) OR (Medicinal Herb[Title/Abstract])) OR (Chinese patent medicine[Title/Abstract])) OR (Chinese patent medicine[Title/Abstract])  #6: #4 OR #5  #7: ((renin–angiotensin system [MeSH Terms]) OR (angiotensin-converting enzyme inhibitor [MeSH Terms])) OR (angiotensin II receptor blocker [MeSH Terms])  #8: #3 AND #6 AND #7 | 82 |
| Web of Science -All Databases | #1: (((((((TS=(Traditional Chinese Medicine)) OR TS=(Chinese Herbal Drugs)) OR TS=(Chinese Plant Extracts Oriental Medicine)) OR TS=(Medicinal Plant)) OR TS=(Pharmaceutical Plant )) OR TS=(Medicinal Herb )) OR TS=(Chinese patent medicine )) OR TS=(Chinese herbal medicine)  #2: TS=(renin–angiotensin system OR angiotensin-converting enzyme inhibitor OR angiotensin II receptor blocker)  #3: TS= (Glomerulonephritides, IgA OR Berger's Disease OR nephropathy, IgA OR IgA Glomerulonephritis OR IgA Nephropathy OR immunoglobulin a nephropathy OR immunoglobulin a nephritis OR IgA nephritis)  #4: #1 AND #2 AND #3 | 632 |
| Embase | #1: 'immunoglobulin A nephropathy'/exp  #2: 'glomerulonephritides, iga':ab,ti OR 'bergers disease':ti,ab,kw OR 'iga nephropathy':ab,ti OR 'immunoglobulin a nephritis':ab,ti OR 'iga nephritis':ab,ti  #3: #1 OR #2  #4: 'Chinese medicine'/exp  #5: 'traditional Chinese Medicine':ab,ti OR 'Chinese Herbal Drugs':ab,ti OR 'Chinese Plant Extracts Oriental Medicine':ab,ti OR 'Medicinal Plant':ab,ti OR 'Pharmaceutical Plant':ab,ti OR 'Medicinal Herb':ab,ti OR 'Chinese patent medicine':ab,ti OR 'Chinese herbal medicine':ab,ti  #6: #4 OR #5  #7: 'renin angiotensin system inhibitor'/exp OR 'angiotensin-converting enzyme inhibitor':ab,ti OR 'angiotensin receptor antagonist'/exp  #8: #3 AND #6 AND #7 | 46 |
| Cochrane | #1: MeSH descriptor: [Medicine, Chinese Traditional] explode all trees  #2: MeSH descriptor: [Drugs, Chinese Herbal] explode all trees  #3: MeSH descriptor: [Medicine, East Asian Traditional] explode all trees  #4: MeSH descriptor: [Plants, Medicinal] explode all trees  #5: #1OR#2OR#3OR#4  #6:(Medicine, Chinese Traditional):ti,ab,kw OR (Drugs, Chinese Herbal):ti,ab,kw OR (Medicine, East Asian Traditional):ti,ab,kw OR (Plants, Medicinal):ti,ab,kw OR (Traditional Chinese Medicine):ti,ab,kw OR (Chinese Drugs, Plant):ti,ab,kw OR (Chinese Herbal Drugs):ti,ab,kw OR (Chinese Plant Extracts):ti,ab,kw OR (Extracts, Chinese Plant):ti,ab,kw OR (Traditional East Asian Medicine):ti,ab,kw OR (Oriental Medicine):ti,ab,kw OR (Medicinal Plant):ti,ab,kw OR (Pharmaceutical Plant):ti,ab,kw OR (Medicinal Herb):ti,ab,kw OR (Chinese patent medicine):ti,ab,kw OR (Chinese herbal medicine):ti,ab,kw  #7: #5OR#6  #8: MeSH descriptor: [Angiotensin-Converting Enzyme Inhibitors] explode all trees  #9: MeSH descriptor: [angiotensin II receptor blocker] explode all trees  #10: (Angiotensin Converting Enzyme Inhibitors):ti,ab,kw OR (Renin Angiotensin System Inhibitor):ti,ab,kw OR (Angiotensin-Converting Enzyme Antagonists):ti,ab,kw OR (ACE Inhibitors):ti,ab,kw OR (Angiotensin I-Converting Enzyme Inhibitors):ti,ab,kw OR (Angiotensin-Converting Enzyme Inhibitor):ti,ab,kw  #11: #8OR#9OR#10  #12: MeSH descriptor: [Glomerulonephritis, IgA] explode all trees  #13: (iga nephropathy):ti,ab,kw OR (immunoglobulin A nephropathy):ti,ab,kw OR (bergers disease):ti,ab,kw OR (immunoglobulin a nephritis):ti,ab,kw OR (iga nephritis):ti,ab,kw  #14:#7AND#11AND#13 | 34 |
| Scopus | #1: (TITLE-ABS-KEY (iga AND nephropathy) OR TITLE-ABS-KEY (immunoglobulin AND a AND nephropathy) OR TITLE-ABS-KEY (glomerulonephritides, AND iga) OR TITLE-ABS-KEY (bergers AND disease) OR Scopus TITLE-ABS-KEY (iga AND nephropathy) OR TITLE-ABS-KEY (immunoglobulin AND a AND nephritis) OR TITLE-ABS-KEY (iga AND nephritis))  #2: (TITLE-ABS-KEY (Medicine, Chinese Traditional) OR TITLE-ABS-KEY (Drugs, Chinese Herbal) OR TITLE-ABS-KEY (Medicine, East Asian Traditional) OR TITLE-ABS-KEY (Plants, Medicinal) OR TITLE-ABS-KEY (Traditional Chinese Medicine) OR TITLE-ABS-KEY (Chinese Drugs, Plant) OR TITLE-ABS-KEY (Chinese Herbal Drugs) OR TITLE-ABS-KEY (Chinese Herbal Drugs) OR TITLE-ABS-KEY (Chinese Plant Extracts) OR TITLE-ABS-KEY ((Extracts, Chinese Plant) OR TITLE-ABS-KEY (Traditional East Asian Medicine) OR TITLE-ABS-KEY (Chinese herbal medicine))  #3: (TITLE-ABS-KEY (Angiotensin-Converting Enzyme Inhibitors) OR TITLE-ABS-KEY (angiotensin II receptor blocker) OR TITLE-ABS-KEY (Angiotensin Converting Enzyme Inhibitors) OR TITLE-ABS-KEY (Renin Angiotensin System Inhibitor) OR TITLE-ABS-KEY (ACE Inhibitors)  #4: #1 AND #2 AND #3 | 66 |
| **CNKI** | TKA=('Traditional Chinese Medicine Science' + 'Medicine, Chinese Traditional' + 'Drugs, Chinese Herbal' + 'Decoctions' + 'Traditional Chinese Medicine' + 'Drugs, Chinese Herbal' + 'decoction') *('IgA nephropathy' + ' iga nephritis ' + ' immunoglobulin A nephropathy '+ ' immunoglobulin A nephritis '+ ' berger’s disease ') AND TKA =('randomized controlled' + 'RCT' + 'Placebo' ) AND TKA =('ACEI' OR 'ARB' ) | 104 |
| **WANFANG DATA** | (The subject word: "Traditional Chinese Medicine Science" OR "Medicine, Chinese Traditional" OR "Drugs, Chinese Herbal" OR "COMPOUNDS(TCD)" OR "Decoctions"）*（The subject word: " IgA nephropathy " OR " iga nephritis " OR " immunoglobulin A nephropathy "）*(abstract: "Randomized controlled" OR "randomized" OR "RCT" OR "Placebo" OR "Clinical Studies" OR "Clinical trials") | 162 |

**Supplementary Table S2.** Node-splitting approach for inconsistency assessment of all comparisons

eGFR

| Side | Direct | | Indirect | | Difference | | P>\|z\| |
| --- | --- | --- | --- | --- | --- | --- | --- |
|  | Coef. | std. Err. | Coef . | std. Err. | Coef. | std. Err. |  |
| A D * | 5.854436 | 3.567274 | -1.737161 | 80.54413 | 7.591597 | 80.62269 | 0.925 |
| B D * | -4.348779 | 2.777732 | -10.61775 | 9.176241 | 6.268971 | 9.665607 | 0.517 |
| B E | -3.994611 | 4.758547 | -.0666861 | 3.845466 | -3.927925 | 6.121414 | 0.521 |
| C D * | -2.272705 | 4.872499 | 14.0443 | 771.809 | -16.317 | 771.8207 | 0.983 |
| C F * | 1.499309 | 24.70472 | -7.420514 | 41.78998 | 8.919823 | 45.44865 | 0.844 |
| D E * | 3.424531 | 1.788969 | -.9053909 | 10.85782 | 4.329922 | 11.06105 | 0.695 |
| D F * | -.4692816 | 25.23769 | 8.448483 | 40.82349 | -8.917764 | 45.44874 | 0.844 |

Scr

| Side | Direct | | Indirect | | Difference | | P>\|z\| |
| --- | --- | --- | --- | --- | --- | --- | --- |
|  | Coef. | std. Err. | Coef . | std. Err. | Coef. | std. Err. |  |
| A C | -1.17e-09 | 14.41523 | -13.16842 | 7.56877 | 13.16842 | 16.28143 | 0.419 |
| A D * | -12.34195 | 5.967765 | -4.204443 | 21.89969 | -8.137508 | 22.31011 | 0.715 |
| B C | 5.155959 | 2.602249 | 6.210008 | 9.723435 | -1.054049 | 10.06602 | 0.917 |
| B D * | 2.995734 | 3.225053 | 6.892726 | 7.26534 | -3.896992 | 7.973553 | 0.625 |
| B F | 1.713847 | 6.118059 | -4.484715 | 3.723395 | 6.198563 | 7.172573 | 0.387 |
| C D* | 1.342371 | 9.490073 | -2.088415 | 3.989849 | 3.430786 | 10.23988 | 0.738 |
| C E * | -6.468884 | 1.868919 | 20.81666 | 152.9001 | 152.9123 | 152.9123 | 0.858 |
| C G * | -.7495146 | 12.22377 | -5.446147 | 26.73875 | 4.696632 | 30.17172 | 0.876 |
| D F * | -6.55601 | 1.471074 | 1.395748 | 12.70318 | -7.951758 | 12.78256 | 0.534 |
| D G * | -1.359514 | 13.78534 | 3.341754 | 24.35436 | -4.701269 | 30.17209 | 0.876 |

24hpro

| Side | Direct | | Indirect | | Difference | | P>\|z\| |
| --- | --- | --- | --- | --- | --- | --- | --- |
|  | Coef. | std. Err. | Coef. | std. Err. | Coef. | std. Err. |  |
| AC | -.5078967 | .3973525 | -.5525667 | .2253467 | .04467 | .4528965 | 0.921 |
| AD* | -.6975668 | .1860474 | -.4903451 | .6024584 | -.2072217 | .6266033 | 0.741 |
| BC | .3276593 | .0865942 | .2619777 | .1800456 | .0656816 | .1997957 | 0.742 |
| BD | .1741018 | .1221193 | .1799017 | .1384751 | -.0057998 | .1847532 | 0.975 |
| BF | -.2628834 | .1910734 | -.2488703 | .1201816 | -.014013 | .225786 | 0.951 |
| CD | -.0243041 | .1519432 | -.2241491 | .1315463 | .1998451 | .2007278 | 0.319 |
| CE* | -.2714293 | .0718845 | 1.095485 | 5.326654 | -1.366914 | 5.327163 | 0.797 |
| CG* | -.4697948 | .1245878 | -.6173635 | .3878498 | .1475687 | .4076277 | 0.717 |
| DF | -.4215142 | .0569847 | -.6055539 | .2679676 | .1840397 | .2739844 | 0.502 |
| DG | -.2900759 | .1655257 | -.4473119 | .2259696 | .157236 | .2806685 | 0.575 |

BUN

| Side | Direct | | Indirect | | Difference | | P>\|z\| |
| --- | --- | --- | --- | --- | --- | --- | --- |
|  | Coef. | std. Err. | Coef. | std. Err. | Coef. | std. Err. |  |
| AC* | -2.839142 | 2.005666 | -.6475688 | 2.794951 | -2.191573 | 3.428224 | 0.523 |
| AD* | -2.853553 | 2.04138 | -1.137553 | 2.897742 | -1.716 | 3.54753 | 0.629 |
| BC | .249499 | .8293895 | .0256475 | 1.978859 | .2238515 | 2.145631 | 0.917 |
| BD | -.0625636 | 1.395591 | .1504612 | 1.630166 | -.2130247 | 2.14596 | 0.921 |
| CD* | -.0475919 | 1.432465 | -.3601767 | 1.581076 | .3125849 | 2.133079 | 0.883 |
| CE* | -.5088272 | .6380193 | 4.985679 | 9.292584 | -5.494506 | 9.314511 | 0.555 |
| CG* | -.04 | 2.036861 | -.2529719 | 4.346505 | .2129719 | 4.763761 | 0.964 |
| DF* | -1.264285 | .4657645 | 5.090477 | 12.23864 | -6.354762 | 12.24753 | 0.604 |
| DG* | .0700002 | 2.07296 | .2889501 | 4.295192 | -.2189499 | 4.763934 | 0.963 |

ADR

| Side | Direct | | Indirect | | Difference | | P>\|z\| |
| --- | --- | --- | --- | --- | --- | --- | --- |
|  | Coef. | std. Err. | Coef. | std. Err. | Coef. | std. Err. |  |
| AD* | -.2529748 | .4619589 | .701342 | 35.77879 | -.9543167 | 35.78177 | 0.979 |
| BC* | .4419354 | .6793118 | -2.802931 | 166.1336 | 3.244866 | 166.1345 | 0.984 |
| CD* | 1.165303 | 1.183245 | -.328428 | 49.6359 | 1.493731 | 49.64951 | 0.976 |
| CE* | -.107948 | .628922 | 2.838863 | 149.5599 | -2.946811 | 149.5613 | 0.984 |
| CG* | .2405353 | 2.8225 | .6962652 | 243.8233 | -2.581965 | 243.8245 | 0.992 |
| DF* | -.1872884 | .3901649 | .531841 | 109.9723 | -.7191294 | 109.9728 | 0.995 |

Notes: A: placebo; B: Traditional Chinese medicine (TCM); C: angiotensin converting enzyme inhibitors (ACEI); D: angiotensin receptor blocker (ARB); E: ACEI+TCM; F:ARB+TCM; G: ACEI+ARB

**Supplementary Table S3. Pooled standardized mean difference and heterogeneity for each direct comparison for frailty.**

| **Comparison** | **Number of RCTs** | **Number of participants** | **(Pooled) SMD (95% CI)** | **I square (%)** | **P value** |
| --- | --- | --- | --- | --- | --- |
| **eGFR** | | | | | |
| ARB vs. placebo | 2 | 335 | 0.484 (0.092,0.876) | 65.6 | 0.088 |
| ARB vs. ACEI | 4 | 124 | -0.046(-0.399,0.308) | 0 | 0.751 |
| ARB vs. TCM | 4 | 1857 | -0.057(-0.150,0.035) | 33.3 | 0.213 |
| ARB vs. TCM+ARB | 8 | 766 | -0.481(-1.073,0.112) | 93.2 | <0.001 |
| ARB vs. ACEI+ARB | 2 | 50 | 0.014(-0.543,0.570) | 0 | 0.876 |
| ACEI vs. ACEI+ARB | 2 | 51 | -0.030(-0.580,0.520) | 0 | 0.915 |
| TCM vs. TCM+ARB | 1 | 269 | 0.178(-0.062,0.417) | NA | NA |
| **Scr** | | | | | |
| ARB vs. placebo | 2 | 242 | -0.848(-1.644, -0.053) | 55.8 | 0.133 |
| ACEI vs. placebo | 1 | 16 | -1.244(-2.327, -0.161) | NA | NA |
| ARB vs. ACEI | 3 | 86 | 0.011(-0.412,0.434) | 0 | 0.995 |
| ARB vs. TCM | 5 | 571 | 0.161 (-0.003, 0.326) | 26.7 | 0.243 |
| ARB vs. TCM+ARB | 23 | 1895 | 0.520(0.258, 0.782) | 86.8 | <0.001 |
| ARB vs. ACEI+ARB | 3 | 90 | 0.024(-0.390, 0.438) | 0 | 0.999 |
| ACEI vs. ACEI+ARB | 3 | 90 | 0.014(-0.400, 0.428) | 0 | 0.991 |
| ACEI vs. TCM | 10 | 694 | 0.238(0.066,0.410) | 22.7 | 0.235 |
| ACEI vs. TCM+ACEI | 15 | 949 | 0.527(0.197,0.856) | 83.6 | <0.001 |
| TCM vs. TCM+ARB | 2 | 331 | 0.151(-0.069,0.371) | 96 | <0.001 |
| **24hpro** | | | | | |
| ARB vs. placebo | 4 | 460 | -0.502(-0.688,-0.317) | 0 | 0.882 |
| ACEI vs. placebo | 1 | 16 | -0.822(-1.848,0.204) | NA | NA |
| ARB vs. ACEI | 6 | 158 | -0.022(-0.337,0.293) | 0 | 0.626 |
| ARB vs. TCM | 6 | 2053 | 0.123(0.036,0.211) | 92.1 | <0.001 |
| ARB vs. TCM+ARB | 25 | 1961 | 1.085(0.727,1.443) | 92.4 | <0.001 |
| ARB vs. ACEI+ARB | 5 | 140 | 0.331(-0.020,0.682) | 83.2 | <0.001 |
| ACEI vs. ACEI+ARB | 7 | 257 | 1.050(0.166,1.933) | 89.7 | <0.001 |
| ACEI vs. TCM | 13 | 901 | 0.579(0.307,0.850) 100.00 | 74.9 | <0.001 |
| ACEI vs. TCM+ACEI | 16 | 1009 | 0.668(0.350,0.987) | 83.2 | <0.001 |
| TCM vs. TCM+ARB | 2 | 331 | 0.786(-0.756,2.329) | 95.9 | <0.001 |
| BUN | | | | | |
| ARB vs. placebo | 1 | 16 | -1.856(-3.053,-0.659) | NA | NA |
| ACEI vs. placebo | 1 | 16 | -2.112(-3.366,-0.858) | NA | NA |
| ARB vs. ACEI | 2 | 46 | -0.036(-0.614,0.543) | 0 | 0.930 |
| ARB vs. TCM | 2 | 119 | 0.033 (-0.327, 0.394) | 45.1 | 0.177 |
| ARB vs. TCM+ARB | 18 | 1282 | 1.009(0.530, 1.488) | 93.6 | <0.001 |
| ARB vs. ACEI+ARB | 1 | 29 | -0.031(-0.763, 0.701) | NA | NA |
| ACEI vs. ACEI+ARB | 1 | 27 | 0.021(-0.734, 0.776) | NA | NA |
| ACEI vs. TCM | 6 | 402 | 0.062(-0.134,0.258) | 0 | 0.935 |
| ACEI vs. TCM+ACEI | 9 | 564 | 0.548(0.033,1.063) | 88.5 | <0.001 |
| ADR | | | | | |
| ARB vs. TCM+ARB | 3 | 194 | 1.667(0.541,5.138) | 0 | 0.882 |
| ARB vs. placebo | 2 | 335 | 0.868(0.424,1.774) | 37.4 | 0.206 |
| ACEI vs. TCM | 2 | 124 | 1.750(0.541,5.662) | 0 | 0.542 |
| ACEI vs. TCM+ACEI | 4 | 272 | 1.263(0.588,2.711) | 0 | 0.968 |
| ACEI vs. ACEI+ARB | 1 | 61 | 2.903(0.320,26.381) | NA | NA |
| ACEI vs. placebo | 1 | 44 | 0.228(0.075, 0.698) | NA | NA |
| ARB vs. TCM | 2 | 1534 | 0.833(0.363,1.914) | 37.7 | 0.205 |

Notes: CI: confidence interval; NA: not applicable; SMD: standardised mean difference.

**Supplementary Table S4.** **Ranking of treatment strategies based on probability of their protective effects.**

| Intervention | eGFR | | Scr | | 24hpro | | BUN | | ADR | |
| --- | --- | --- | --- | --- | --- | --- | --- | --- | --- | --- |
|  | SUCRA % | Rank | SUCRA % | Rank | SUCRA % | Rank | SUCR A% | Rank | SUCRA % | Rank |
| placebo | 10.7 | 6 | 4.4 | 7 | 0 | 7 | 8.8 | 7 | 17.8 | 7 |
| TCM | 78.0 | 1 | 67.2 | 3 | 62.3 | 3 | 51.4 | 3 | 79.2 | 1 |
| ACEI | 56.9 | 3 | 29.4 | 6 | 18 | 6 | 42.5 | 6 | 63.6 | 3 |
| ARB | 35.9 | 5 | 39.4 | 5 | 35 | 5 | 48.3 | 5 | 29.6 | 6 |
| ACEI+TCM | / | / | 76.1 | 2 | 54.4 | 4 | 62.5 | 2 | 67.2 | 2 |
| ARB+TCM | 67.3 | 2 | 85.7 | 1 | 95.2 | 1 | 87.6 | 1 | 39.4 | 5 |
| ACEI+ARB | 51.2 | 4 | 47.9 | 4 | 85.1 | 2 | 48.8 | 4 | 53.1 | 4 |
