# Supplementary material for: Efficacy of traditional Chinese medicine versus angiotensin-converting enzyme inhibitors, angiotensin receptor blockers, and their combinations in the treatment of IgA nephropathy: a systematic review and network meta-analysis
Source: Front Pharmacol. 2024 Mar 21;15:1374377. doi: 10.3389/fphar.2024.1374377 (PMC10991836; doi:10.3389/fphar.2024.1374377)
Supplement: Supplementary file 2 [file Table2.DOCX]

Supplementary Material

**Supplementary Table** 1. Characteristics of clinical trials and patients

| Study | No of patients | | Sex (male %) | | Age (years) | | Treatment | | Duration  (months) | GFR (mL/min/1.73 m^2^) | | Serum creatinine (μmol/L) | | 24hpro (g/d) | |
| --- | --- | --- | --- | --- | --- | --- | --- | --- | --- | --- | --- | --- | --- | --- | --- |
|  | T | C | T | C | T | C | T | C |  | T | C | T | C | T | C |
| Shima et al. (2019) | 31 | 31 | 58.1 | 51.6 | 12.0 ± 3.2 | 11.9 ± 3.3 | ACEI+ARB | ACEI | 24 | 121.3 ± 15.3 | 120.1±13.8 |  |  |  |  |
| Li et al. (2020) | 735 | 735 | 48 | 46.3 | 37.7±10.9 | 37.1±10.4 | TCM | ARB | 12 | 94.2 ± 24.4 | 95.7±24.0 | 80.3±25.2 | 81.8±25.1 | 1.24 ± 0.67 | 1.21±0.64 |
| Li et al. (2006) | 54 | 55 | 24.0 | 30.9 | 40±10 | 41±9 | ARB | placebo | 36 | 85.9± 37.30 | 72.47±35.16 | 114.04±47.74 | 98.12±42.43 | 1.80±1.24 | 2.35±1.71 |
| Zhang et al. (2014) | 135 | 133 | 72 | 67 | 38.1±12.7 | 37.3±12.5 | ARB | TCM | 6 | 106±23 | 108±24 | 70.72±19.45 | 72.49±18.56 | 1.08±0.45 | 1.05±0.42 |
| Zhang et al. (2014) | 136 | 133 | 64 | 67 | 37.1±11.1 | 37.3±12.5 | ARB+TCM | TCM | 6 | 106±24 | 108±24 | 71.60±20.33 | 72.49±18.56 | 1.07±0.44 | 1.05±0.42 |
| Nakamura et al. (2007) | 8 | 8 | 62.5 | 50 | 34±7 | 31±8 | ARB | ACEI | 3 |  |  | 88.4±26.52 | 97.24±17.68 | 2.1±0.6 | 1.9±0.3 |
| Nakamura et al. (2007) | 8 | 8 | 62.5 | 50 | 34±7 | 31±7 | ARB | ARB+ACEI | 3 |  |  | 88.4±26.52 | 97.24±17.68 | 2.1±0.6 | 2.0±0.4 |
| Horita et al. (2004) | 10 | 10 | 50 | 40 | 42.7±12 | 39.6±10.8 | ARB | ACEI | 6 | 88.3±62.61 | 92.5 ±54.39 | 77.79±47.53 | 75.14±58.69 | 0.81±1.39 | 0.73±1.14 |
| Horita et al. (2004) | 10 | 11 | 50 | 45.5 | 42.7±12 | 39.6±10.4 | ARB | ARB+ACEI | 6 | 88.3±62.61 | 91.5±81.59 | 77.79±47.53 | 73.37±65.67 | 0.81±1.39 | 0.75±0.99 |
| Horita et al. (2006) | 16 | 14 | 56.3 | 66.7 | 42±9 | 43±10 | ARB | ACEI | 12 | 88.0 ± 72.8 | 89.8 ±85.68 | 78.68 ± 46.32 | 74.26±56.24 | 0.83 ± 1.76 | 0.60 ± 0.79 |
| Horita et al. (2006) | 16 | 13 | 56.3 | 53.8 | 42±9 | 38±9 | ARB | ARB+ACEI | 12 | 88.0 ± 72.8 | 95.3 ± 80.76 | 78.68 ± 46.32 | 74.26±63.64 | 0.83 ± 1.76 | 0.80 ± 1.19 |
| Renke et al. (2004) | 18 | 18 | 38.9 | 66.7 | 40.4±11.9 | 43.4±10.1 | ARB | ACEI | 9 |  |  |  |  | 2.17±1.52 | 2.6±1.69 |
| Renke et al. (2004) | 18 | 16 | 38.9 | 68.8 | 40.4±11.9 | 37.7±12.7 | ARB | ARB+ACEI | 9 |  |  |  |  | 2.17±1.52 | 3.25±1.82 |
| Shen et al. (2012) | 112 | 114 | 51.8 | 49.1 | 50.2±10.4 | 49.1± 11.5 | ARB | placebo | 12 | 44.8 ± 8.1 | 44.8 ± 8.5 | 130.03±23.87 | 131.72±24.75 | 1.72±0.47 | 1.73±0.49 |
| Liang et al. (2017) | 26 | 26 | 53.85 | 46.15 | 38.7±12. | 37.1±11.7 | ARB | ARB+TCM | 6 | 104 ± 25 | 105 ± 23 | 73.4 ±19.5 | 75.1 ± 22.1 | 1.09±0.45 | 1.07±0.43 |
| Zhao (2015) | 30 | 30 | 50 | 53.3 | 32±6.32 | 32±6.32 | ARB | ARB+TCM | 3 |  |  | 88.98±12.50 | 92.46±13.71 | 1.67±0.25 | 1.70±0.25 |
| Su and Zhang (2014) | 24 | 24 | 54.17 | 50 | 34.2±8.1 | 34.4±7.9 | ARB | ARB+TCM | 3 | 95.6±5.4 | 96.2±5.3 |  |  | 1.92±0.66 | 1.85±0.77 |
| Wang et al. (2014) | 78 | 78 | 51.28 | 57.69 | 39.4±6.2 | 37.3±5.7 | ARB | ARB+TCM | 12 |  |  | 102.1±30.3 | 115.6±27.8 | 2.47±0.53 | 2.54±0.41 |
| Xiang et al. (2014) | 30 | 30 | 60 | 63.33 | 51.3±8.2 | 50.3±9.6 | ARB | ARB+TCM | 3 |  |  | 92.15± 12.03 | 89.73±12.14 | 2.09±0.53 | 1.19±0.45 |
| Duan and Wang (2014) | 30 | 30 | 36.67 | 40 | 33±8 | 35±9 | ARB | ARB+TCM | 6 |  |  |  |  | 1.35±0.66 | 1.46±0.70 |
| Xue (2012) | 30 | 30 | 60 | 66.67 | 31.77±10.47 | 32.87±11.33 | ARB | ARB+TCM | 3 |  |  | 85.87±20.55 | 80.70+21.11 | 1.005±0.83 | 1.055±0.86 |
| Li et al. (2012) | 30 | 30 | 56.67 | 53.33 | 34.5±11.7 | 32.8±12.7 | ARB | ARB+TCM | 3 |  |  | 86.4±25.4 | 87.4±26.6 | 1.35±0.63 | 1.48±0.51 |
| Guo et al. (2010) | 35 | 35 | 52.29 | 62.86 | 28.86±8.95 | 30.60±8.96 | ARB | TCM | 12 |  |  | 91.41±11.67 | 89.59±8.57 | 1.44±0.27 | 1.52±0.35 |
| Zhou and Yao (2010) | 30 | 30 | 56.67 | 53.33 | 32.8±12.6 | 33.2±11.8 | ACEI | TCM | 12 |  |  | 85.87±42.96 | 86.66±43.3 | 2.40±1.12 | 2.45±1.01 |
| Wang (2020a) | 17 | 17 | 58.82 | 64.71 | 43.75± 3.92 | 42.61± 4.22 | ARB | ARB+TCM | 6 |  |  | 138.08±13.51 | 137.15±12.66 | 1.73±0.60 | 1.71±0.45 |
| Wei (2019) | 35 | 35 | 60 | 54.29 | 37.65±5.58 | 39.57±5.16 | ARB | ARB+TCM | 3 |  |  | 108.36±7.27 | 108.91±7.36 |  |  |
| Cai (2018) | 34 | 34 | 52.94 | 55.88 | 46.12±9.05 | 45.78±8.83 | ARB | ARB+TCM | 6 | 105.12±12.93 | 104.29±13.06 | 136.36±14.34 | 134.21±14.34 | 1.68±0.48 | 1.70±0.51 |
| Xu (2020) | 29 | 29 | 58.62 | 55.17 | 40.03±2.49 | 39.65±2.81 | ACEI | ACEI+TCM | 3 |  |  | 110.20±8.05 | 109.59±7.63 | 1.68±0.69 | 1.71±0.64 |
| Hou et al. (2009) | 30 | 30 | 60 | 60 | 34.5±13.4 | 38.6±14.2 | ACEI | ACEI+ARB | 3 |  |  |  |  | 2.2±0.6 | 2.2±0.6 |
| Liu et al. (2007b) | 20 | 20 | 70 | 65 | 13-58 | 14-61 | ARB | ACEI | 6 |  |  | 126.23±74.24 | 126.78±72.04 | 2.21±1.43 | 2.18±1.36 |
| Liu et al. (2007b) | 20 | 21 | 70 | 76.19 | 13-58 | 13-54 | ARB | ACEI+ARB | 6 |  |  | 126.23±74.24 | 126.06±73.14 | 2.21±1.43 | 2.16±1.42 |
| Chang et al. (2021a) | 29 | 29 | 44.83 | 48.28 | 39.03±9.14 | 37.34±6.77 | ARB | TCM | 4 |  |  | 90.66±28.5 | 87.88±23.12 | 1.75±0.54 | 1.6±0.46 |
| Wang (2021a) | 27 | 25 | 48.19 | 52 | 39.96±9.29 | 37.84±6.55 | ARB | TCM | 6 |  |  | 93.29±26.37 | 96.2±25.91 | 1.68±0.46 | 1.51±0.49 |
| Long (2021) | 30 | 30 | 43.3 | 36.67 | 40.40±12.73 | 41.87±13.11 | ACEI | ACEI+TCM | 3 |  |  | 67.87±9.36 | 65.40±9.26 | 1.50.±0.22 | 1.50±0.29 |
| Lu and Ma (2021) | 30 | 30 | 46.67 | 50 | 41.00± 11.69 | 42.27±1 1.46 | ARB | ACEI+ARB | 3 |  |  | 104.29±40.1 | 106.8±42.85 | 1.91±0.92 | 1.85±1.17 |
| Guo and Du. (2023) | 50 | 50 | 54 | 52 | 41.16±3.33 | 42.06±3.84 | ARB | ARB+TCM | 6 |  |  | 82.32±4.51 | 81.65±3.65 |  |  |
| Guan et al. (2005) | 30 | 32 | 53.3 | 53.13 | 30.4±6.2 | 31.2±6.8 | TCM | ARB+TCM | 3 |  |  | 212.8±60.4 | 216.4±56.2 | 2.24±1.32 | 2.26±1.27 |
| Wang et al. (2010a) | 32 | 32 | 53.13 | 53.13 | 31.30±9.75 | 31.10±9.83 | ACEI | TCM | 3 |  |  | 88.5±20.8 | 85.2±29.0 | 1.47±0.82 | 1.48±0.84 |
| Han and Qiu (2010) | 26 | 29 | 53.85 | 51.72 | 38±16.3 | 37±17.4 | ACEI | ACEI+TCM | 3 |  |  | 95.5±7.6 | 98.7±9.7 | 1.93±0.93 | 1.89±0.58 |
| Huang et al. (2008b) | 35 | 35 | 24 | 22 | 28.23±10.32 | 27.63±15.55 | ACEI | TCM | 6 |  |  |  |  | 0.83±0.56 | 0.86±0.42 |
| Liu et al. (2010) | 29 | 32 | 58.62 | 65.63 | 32.72±8.76 | 31.40±8.74 | ACEI | TCM | 4 |  |  |  |  | 1.372±0.372 | 1.324±0.396 |
| Liu et al. (2007a) | 32 | 32 | 53.13 | 53.13 | 36.0±12.0 | 36.0±12.0 | ACEI | ACEI+TCM | 6 |  |  | 123.3±46.4 | 121.1±47.2 | 2.1±1.4 | 2.0±1.4 |
| Gao (2009) | 29 | 30 | 48.28 | 60 | NA | NA | ACEI | ACEI+TCM |  |  |  | 75.02±18.22 | 74.44±19.76 | 1.44±0.55 | 1.34±0.46 |
| Zhang et al. (2019a) | 40 | 40 | NA | NA | NA | NA | ARB | TCM | 6 |  |  | 110.98±35.93 | 113.95±39.60 | 1.19±0.53 | 1.34±0.75 |
| Pan et al. (2017) | 31 | 31 | 61.29 | 54.39 | 31.4±12.3 | 33.8±1.9 | ARB | ARB +TCM | 3 |  |  | 88.23 ± 35.34 | 86.57 ± 32.89 | 1.38 ± 0.46 | 1.34 ± 0.56 |
| Huang and He (2011) | 17 | 33 | 58.82 | 54.55 | NA | NA | ACEI | ACEI+TCM | 3 |  |  | 135.78±49.15 | 136.12±50.57 | 2.71±0.42 | 2.69±0.47 |
| Luo et al. (2023) | 40 | 40 | 45 | 35 | 48. 95 ±7. 26 | 47. 84 ± 6.35 | ACEI | ACEI+TCM | 3 |  |  | 128.39±5.62 | 132.41±6.55 | 2.39±0.62 | 2.41±0.5 |
| Wei et al. (2017) | 30 | 30 | 63.33 | 56.67 | 36.31±8.96 | 36.26±8.91 | ACEI | TCM | 3 |  |  | 67.68±11.46 | 63.53±10.44 | 0.71±0.43 | 0.72±0.45 |
| Chen et al. (2022) | 30 | 30 | 56.67 | 53.33 | 37.5±10.64 | 38.27±10.72 | ARB | ARB +TCM | 3 |  |  | 70.56±11.21 | 71.37±11.02 | 2.13±0.31 | 2.05±0.32 |
| Xu et al. (2009) | 25 | 27 | 44 | 48.15 | 39.2±12.39 | 36.8±12.17 | ACEI | TCM | 6 |  |  | 91.56±30.68 | 83.41±22.37 | 1.12±0.44 | 1.32±0.50 |
| Huang (2008a) | 30 | 30 | 60 | 56.67 | NA | NA | ACEI | ACEI+TCM | 3 |  |  | 74.23±19.29 | 76.07±20.36 | 1.39±0.67 | 1.29±0.57 |
| Wang et al. (2021c) | 45 | 45 | 57.78 | 55.56 | 52.43±13.11 | 54.38±11.25 | ARB | ARB +TCM | 6 |  |  | 78.78±19.95 | 77.24±20.32 | 2.11±0.49 | 2.16±0.58 |
| Wang et al. (2020d) | 35 | 35 | 51.43 | 57.14 | 53.48 ± 11.75 | 51.63 ± 12.51 | ARB | ARB +TCM | 6 |  |  | 79.34±20.93 | 81.03±20.62 | 2.14±0.56 | 2.24±0.61 |
| Gan et al. (2019) | 39 | 39 | 51.28 | 60 | 32.95±3. 26 | 33.27±3. 31 | ARB | ARB +TCM | 3 |  |  |  |  | 2.90±0.28 | 2.88±0.27 |
| Han et al. (2016) | 50 | 50 | 40 | 44 | 34.36 ± 8.05 | 35.28± 8.24 | ACEI | ACEI+TCM | 6 |  |  | 92.47±17.38 | 90.25±18.65 | 2.15±0.77 | 2.03±0.75 |
| Zhao et al. (2021a) | 40 | 40 | 57.5 | 55 | 37.9±11.6 | 35.8±10.8 | ARB | ARB +TCM | 6 |  |  | 93.36±12.74 | 95.90±10.30 | 1.31±0.11 | 1.34±0.25 |
| Chen et al. (2007) | 66 | 65 | 60.60 | 56.92 | 33.65±12.36 | 33.98±11.05 | ACEI | TCM | 4 |  |  | 87.19±40.21 | 79.42±31.29 | 1.40±0.98 | 1.32±0.87 |
| Chen et al. (2006) | 34 | 36 | 38.24 | 50 | 30.97±8.67 | 30.33±12.26 | ACEI | TCM | 4 |  |  | 68.47±19.41 | 71.80±35.74 | 1.17±0.76 | 1.22±0.86 |
| Xiang et al. (2009) | 32 | 32 | 53.13 | 56.25 | 37.26±19.66 | 32.56±13.16 | ACEI | ACEI+TCM | 3 |  |  | 142.08±30.98 | 139.86±33.27 | 1.91±0.97 | 1.87±1.05 |
| Huang et al. (2012) | 25 | 25 | NA | NA | NA | NA | ACEI | ACEI+TCM | 3 |  |  | 72.67±13.45 | 72.67±13.45 | 2.03±0.67 | 2.21±1.01 |
| Wang and Shou (2010b) | 28 | 32 | NA | NA | NA | NA | ACEI | ACEI+ARB | 6 |  |  |  |  | 2.3±0.5 | 2.3±0.5 |
| Li et al. (2018) | 38 | 39 | 34.21 | 35.90 | 42. 20 ±10. 89 | 42. 08 ±9. 71 | ACEI | ACEI+TCM | 3 |  |  | 90.94±33.72 | 90.88±34.81 | 1.15±0.74 | 1.23±0.77 |
| Yang and Chen (2010) | 21 | 21 | NA | NA | NA | NA | ACEI | ACEI+TCM | 3 |  |  | 99.45±37.83 | 98.26±38.72 | 1.48±0.66 | 1.51±0.65 |
| Pan et al. (2022) | 60 | 60 | 32 | 34 | 32.6 ± 6.9 | 30.6± 8.9 | ARB | TCM | 6 |  |  | 74.08±16.08 | 75.33±15.79 | 1.23±0.33 | 1.18±0.30 |
| Meng et al. (2015) | 43 | 44 | 23 | 24 | 38.74±11.67 | 40.56±11.71 | ACEI | TCM | 12 |  |  | 91.46±34.28 | 92.34±36.22 | 1.99±0.51 | 2.08±0.42 |
| Han (2012) | 26 | 26 | 34.62 | 42.31 | NA | NA | ARB | ARB+TCM | 3 |  |  | 95.8±136 | 98.7±143.4 | 1.68±1.18 | 1.57±0.94 |
| Zhang et al. (2016) | 30 | 30 | 66.67 | 70 | 32. 00 ± 6. 06 | 33. 00 ± 9. 53 | ACEI | TCM | 18 |  |  | 86.14±18.69 | 88.02±17.55 | 0.74±0.18 | 0.78±0.14 |
| Wang et al. (2018) | 25 | 25 | 52 | 56 | 32.3 ± 3.9 | 31.9 ± 3.6 | ARB | ARB+TCM | 3 | 68.67±18.74 | 67.41±19.54 |  |  | 2.33±0.43 | 2.31±0.41 |
| Zhao et al. (2022) | 48 | 49 | 54.17 | 51.02 | 32.75±3.08 | 33.03 ±2.77 | ARB | ARB+TCM | 3 | 61.74±6.22 | 61.83±6.14 | 95.48±9.26 | 95.62±9.15 | 1.78±0.25 | 1.70±0.29 |
| Wang et al. (2020c) | 39 | 40 | 43.59 | 37.5 | 43. 82 ± 12. 65 | 43. 55 ± 12. 35 | ARB | TCM | 6 | 82.09±26.07 | 81.00±25.18 | 91.56±35.42 | 91.13±32.88 | 1.36±0.67 | 1.49±0.63 |
| Zhang et al. (2019b) | 20 | 20 | 55 | 45 | 43. 40 ± 11. 31 | 45. 95 ± 10. 35 | ARB | TCM | 6 | 72. 55 ± 29. 43 | 67. 80 ± 26. 85 | 110.98±35.93 | 113.95±39.60 |  |  |
| Yao and Zhou (2011) | 32 | 32 | 56.25 | 53.13 | NA | NA | ACEI | ACEI +TCM | 3 |  |  | 88.5±20.8 | 85.2±29.0 | 1.47±0.82 | 1.48±0.84 |
| Wang et al. (2020b) | 61 | 61 | 47.54 | 50.82 | 41.55±14.17 | 42.54±14.28 | ARB | ARB+TCM | 6 | 68.89±3.59 | 64.84±3.54 | 116.67±6.23 | 119.55±6.82 | 0.99±0.10 | 1.13±0.12 |
| Wang et al. (2019) | 78 | 78 | 47.43 | 51.28 | 42.71±10.41 | 42.67±10.47 | ARB | ARB+TCM | 6 | 69.77±3.04 | 67.37± 2.81 | 116.60± 5.25 | 116.79±5.14 | 1.10±0.09 | 1.23±0.09 |
| Guo (2019) | 30 | 30 | 50 | 46.67 | 35.93±12.99 | 35.50±11.73 | ARB | ARB+TCM | 3 |  |  | 69.33±14.28 | 75.53±16.57 | 1.50±0.76 | 1.66±0.76 |
| Li et al. (2023) | 35 | 38 | 54.26 | 55.26 | 37.18±7.24 | 36.42±8.89 | ARB | ARB+TCM | 3 |  |  | 88.84 ± 5.52 | 89.21 ± 4.47 | 3.78 ± 0.58 | 3.74 ± 0.53 |
| Jia (2012) | 33 | 33 | NA | NA | NA | NA | ACEI | ACEI +TCM | 3 |  |  | 85.86±5.46 | 85.05±3.18 | 1.92±0.95 | 1.81±1.04 |
| Duan (2011) | 30 | 30 | 23.33 | 33.33 | NA | NA | ACEI | ACEI +TCM | 3 |  |  |  |  | 1.44±0.73 | 1.49±0.77 |

T, treatment; C, control.
